# Hot-carrier cooling and photoinduced refractive index changes in organic–inorganic lead halide perovskites

**DOI:** 10.1038/ncomms9420

**Published:** 2015-09-25

**Authors:** Michael B. Price, Justinas Butkus, Tom C. Jellicoe, Aditya Sadhanala, Anouk Briane, Jonathan E. Halpert, Katharina Broch, Justin M. Hodgkiss, Richard H. Friend, Felix Deschler

**Affiliations:** 1 Cavendish Laboratory, Department of Physics, JJ Thomson Avenue, Cambridge, CB3 0HE, United Kingdom; 2 The MacDiarmid Institute for Advanced Materials and Nanotechnology, and School of Chemical and Physical Sciences, Victoria University of Wellington, Wellington 6140, New Zealand

## Abstract

Metal-halide perovskites are at the frontier of optoelectronic research due to solution processability and excellent semiconductor properties. Here we use transient absorption spectroscopy to study hot-carrier distributions in CH_3_NH_3_PbI_3_ and quantify key semiconductor parameters. Above bandgap, non-resonant excitation creates quasi-thermalized carrier distributions within 100 fs. During carrier cooling, a sub-bandgap transient absorption signal arises at ∼1.6 eV, which is explained by the interplay of bandgap renormalization and hot-carrier distributions. At higher excitation densities, a ‘phonon bottleneck' substantially slows carrier cooling. This effect indicates a low contribution from inelastic carrier-impurity or phonon–impurity scattering in these polycrystalline materials, which supports high charge-carrier mobilities. Photoinduced reflectivity changes distort the shape of transient absorption spectra and must be included to extract physical constants. Using a simple band-filling model that accounts for these changes, we determine a small effective mass of *m*_r_*=*0.14 *m*_o_, which agrees with band structure calculations and high photovoltaic performance.

## Abstract (web summary)

The use of organic–inorganic metal-halide perovskites in hot-carrier devices depends on deepening the understanding of photoexcitations in these materials. Here, Price *et al.* use transient absorption spectroscopy to study hot-carrier distributions in CH_3_NH_3_PbI_3_ and quantify key semiconductors parameters.

## Graphical abstract

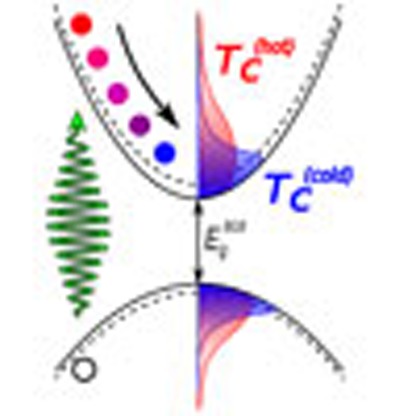

Optoelectronic research has witnessed a resurgence of interest in organic–inorganic metal-halide perovskites. Since the first reports of high photovoltaic efficiencies^1^,^2^in 2012, solar cells with over 20% power conversion efficiency have been obtained^3^,^4^, along with light-emitting diodes^5^ and optically pumped lasing structures^6^,^7^,^8^. Recently, optical and dielectric constants of perovskites and their importance for efficient photovoltaic devices have been reported from steady-state electro-optical measurements^9^. Progress towards future applications such as hot-carrier photovoltaics^10^, electrically pumped lasers^11^,^12^ and NOR flash memory^13^ depends on deepening the understanding of photoexcitations in these materials, particularly hot-carrier dynamics. The interactions of charge carriers with phonons determine their relaxation to the band edge on photoexcitation^14^ and their behaviour under large fields^15^,^16^. The importance of carrier thermalization in perovskites is highlighted in the recent transient spectroscopy investigation by Chen *et al.*,^17^ who showed that the temporal onset of amplified spontaneous emission was dependent on the relaxation of carriers to the band edge. Characterizing charge transport in these materials also relies on parameters accessed through transient optical spectroscopy. Despite the importance of charge transport to photovoltaic device efficiencies, direct transport measurements remain challenging due to the difficulty of fabricating doped and gated device structures, and to interference from effects such as ion drift^18^.

Important spectroscopically accessible parameters for the quantification of semiconductor performance are carrier scattering times and the effective masses of electrons and holes. Transient absorption (TA) spectroscopy is well suited to interrogate the optoelectronic properties of these materials and has already advanced the field^19^,^20^,^21^. Important processes, such as hot-carrier cooling, should be manifest in TA spectra, but uncertainty over the origin of TA signals has so far limited interpretation. In particular, the long-lived high-energy photoinduced absorption (PIA) above the band edge (>1.7 eV) and the short-lived derivative feature just below the bandgap at 1.58 eV are not yet understood^6^,^17^.

Here we show that above bandgap, non-resonant excitation creates quasi-thermalized carrier distributions, which show reduced cooling rates at high fluence due to a phonon bottleneck. To quantify relevant parameters, such as carrier cooling rates and effective masses, we provide a complete spectral analysis of TA spectra from femto- to nanosecond timescales. We find that photoinduced reflectivity changes distort the shape of the TA spectra and must be included to extract physical constants. Using a simple band-filling model, we determine a small effective mass of *m*_r_*=*0.14 *m*_o_.

## Results

### Excitation-dependent carrier cooling due to hot phonon effect

We investigate the hot-carrier properties in CH_3_NH_3_PbI_3_ at 300 K using TA measurements on a timescale of 100 s of femtoseconds, which allows us to resolve the dynamics of hot-carrier distributions via the spectral shape of the ground-state bleaching signal above the band edge. We prepare our thin film samples (thickness ∼100 nm) as described elsewhere^22^, by mixing 3:1 molar stoichiometric ratios of CH_3_NH_3_I and Pb(CH_3_COO)_2_ in *N*,*N*-dimethylformamide with 20 wt% concentration^22^, spin coating in an inert nitrogen environment and annealing in air. We obtain uniform films with small crystal sizes (Supplementary Fig. 1), which leads to a reduction in pump light scattering and allows TA measurements with excitation close to the probing region at the band edge. Femtosecond TA measurements are performed as described in ref. 6.

**Figure 1:**
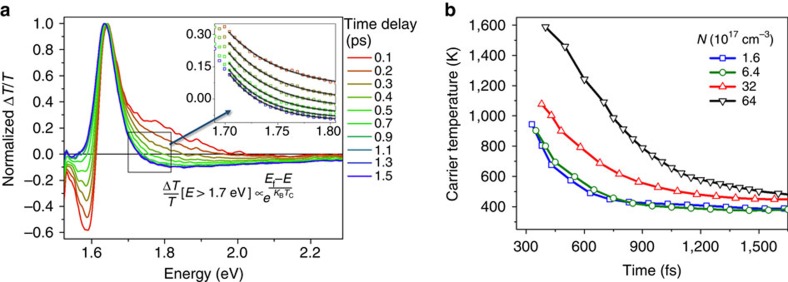
TA spectra showing hot photoexcited carrier distributions and cooling. (**a**) Normalized transient absorption (TA) spectra of CH_3_NH_3_PbI_3_, pumped at 2.25 eV (pump resolution ∼200 fs), with an initial (*t*=0) average carrier density of *N*=6.4 × 10^17^ cm^−3^ over the illuminated area. Spectral broadening at early times (before 2.5 ps) indicated hot-carrier distributions. Inset: cooling rates were obtained from a global fit of the high-energy tail of each timeslice between *E*=1.7 and 1.85 eV to a Boltzman distribution. (**b**). Change in photoexcited carrier temperatures against time delay for different initial (*t*=0) carrier densities, obtained from fits as described in Fig. 1a. At densities below ∼6 × 10^17^ cm^−3^, starting from an initial temperature of ∼1,000 K down to near room temperature, the carriers cool with a principal rate constant of ∼ 230 fs. At higher carrier densities, there is a slowing of the cooling rate consistent with a hot phonon bottleneck effect, which agrees with a much higher absolute carrier temperature at short-time delays (∼300 fs).

Figure 1a shows normalized TA spectra of CH_3_NH_3_PbI_3_ films after photoexcitation at 2.25 eV with an initial (*t*=0 s) average carrier density of *N*=6.4 × 10^17^ cm^−3^ over the illuminated area—determined from the optical density (from reflection-corrected ultraviolet–visible measurements), the film thickness (from cross-sectional scanning electron microscope imaging) and the incident pump fluence. We find that at early times after photoexcitation, before ∼5 ps, the high-energy tail of the spectrum is broadened, and a negative TA feature is present just below the bandgap (at ∼1.58 eV). We attribute the broadening to the presence of a quasi-equilibrium carrier distribution at a temperature *T*_c_, which is higher than the lattice temperature of the sample^14^,^23^. This carrier temperature represents an average of both electron- and hole-carrier distribution temperatures. The absence of spectral signatures associated with non-thermalized carrier distributions, such as spectrally narrow features above the bandgap, leads us to conclude that, at room temperature, thermalization to quasi-equilibrium carrier distributions occurs within the temporal resolution of our experiment, on a timescale of 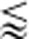
100 fs. Once quasi-equilibrium is reached for the electron–hole plasma, an estimate of the carrier temperature can be obtained from fits to the spectra above the band edge (Fig. 1a, inset). For this, we assume that (1) the TA signal is proportional to the change in absorption coefficient, Δ*T*/*T*∝Δ*α*, (2) the hot-carrier distribution follows a Boltzmann distribution and (3) the density of states is approximately constant in the analysed region. To ensure we only study carrier distributions with a defined temperature, values are extracted after 300 fs.

Figure 1b shows that for densities below ∼6 × 10^17^ cm^−3^, carriers cool within the first 2 ps with a decay constant of 230 fs. At higher carrier densities, we see a clear retardation of the decay rate—from a time constant of 230 fs at 6 × 10^17^ cm^−3^ to 770 fs at 60 × 10^17^ cm^−3^ —along with a departure from a mono-exponential decay and much higher initial carrier temperatures. We attribute this decrease in the cooling rate to a ‘hot phonon bottleneck effect'. This is commonly observed in inorganic crystalline semiconductors^24^,^25^ and arises when there is substantial carrier reabsorption of optical phonons. After 2 ps, a slower second decay becomes apparent. This slower decay is the reason the cooling curves in Figure 1b have not fully decayed to the lattice temperature (near 300K) by 1500, fs. This slow component becomes more apparent when carriers are excited closer to resonance with the bandgap (Supplementary Fig. 2). This behaviour is consistent with an initial cooling process dominated by carrier interactions with optical phonons and suggests two principle cooling decay components. This is likely a manifestation of the multiple phonon modes predicted to be present in these materials^26^. We posit a dominant LO phonon emission (the energy of which is ∼13 meV for pure lead iodide^27^, but calculated to be lower for the studied perovskite lead iodides^26^) that is responsible for the fast decay, while the slow decay is caused by other phonon emission pathways. These can either be the delayed emission of LO phonons or emission of acoustic/optical phonons with energy below the ones causing the fast dominant decay. We note that at significantly lower excitation fluences, additional interactions of charge carriers—for example, with defects—might occur. We do not expect that Auger processes have significant effects at the excitation densities measured, as the band edge bleach kinetics show no significant decays on the timescale of the cooling.

The timescale of carrier cooling can be related to parameters that determine charge transport. In general, carrier mobilities are reduced by strong impurity scattering, which also results in accelerated carrier cooling. Thus, slow carrier cooling is observed here, which lasts many picoseconds, is characteristic of high mobility semiconductors such as GaAs. While carrier cooling is faster for CH_3_NH_3_PbI_3_, it is still surprisingly slow, given the presence of heavy lead atoms, as well as the large number of available phonon modes, and the disordered polycrystalline nature of the material (crystallite size ∼100 nm). The presence of a hot phonon bottleneck, which allows hot carriers to persist for many picoseconds, highlights the limited contribution of phonon-impurity scattering, which in turn enables high carrier mobilities.

### Interplay of hot-carrier effects and bandgap renormalization

**Figure 2:**
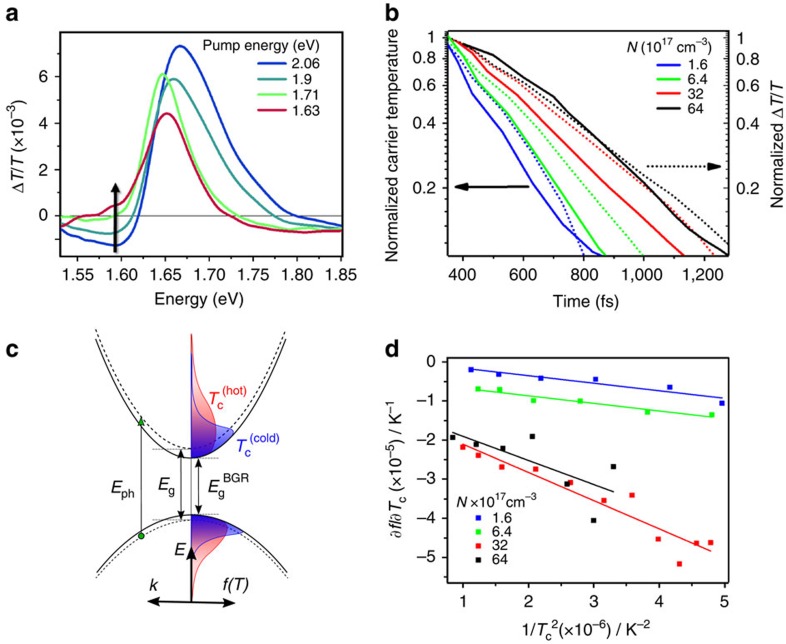
Effect of carrier cooling, bandgap renormalization and Burstein–Moss effect. (**a**) Transient absorption spectra of CH_3_NH_3_PbI_3_ for a series of pump energies, taken at 250 fs after pump excitation (carrier densities ∼2 × 10^17^ cm^−3^). As the pump energy is moved towards resonance with the band edge, the negative feature at ∼1.58 eV reduces (blue arrow). Under resonant pump energy with the band edge (1.63 eV, full-width at half-maximum=25 meV), the negative feature completely disappears. (**b**) Normalized kinetics of the negative TA signal at 1.58 eV (dashed lines), excited at 2.25 eV, compared with normalized carrier cooling kinetics (solid lines) at carrier densities stated. With increasing carrier density, the TA kinetics of the 1.58 eV feature follow the same trend as the carrier cooling rates, that is, higher densities give slower decays. The normalized cooling curves show a consistently faster decay than the decay of the 1.58 eV TA feature. (**c**) A schematic representation showing the relationship between carrier cooling and bandgap renormalization. Carriers are excited into (out of) conduction (valence) bands, photoexcited carriers interact and bandgap renormalization moves the bandgap from *E*_g_^0^ to *E*_g_^BGR^. ‘Hot' photoexcited carrier distributions (red) with high carrier temperatures occupy fewer states at the new band edge, which gives rise to the PIA at 1.58 eV. As carrier temperature reduces (blue), increased occupation of the renormalized states leads to a reduction of the PIA. (**d**) Change in occupation at the band edge ∂*f* (taken from the transient absorption kinetic at 1.58 eV) divided by the change in carrier temperature ∂*T_c_*, plotted versus 1/*T_c_*^2^. Here we present the same data as Fig. 2b (before normalization), but with time plotted as an intrinsic variable. ∂*f*/∂*T_c_* is obtained by taking the derivative of the transient absorption kinetic at 1.58 eV with respect to time −*∂f*/*∂t*, and dividing this by the change in the carrier temperature *T*_c_ with respect to time −∂*T_c_*/∂*t*. The data are well described by linear fits (solid lines), which indicates that ∂*f*/∂*T_c_* is proportional to 1/*T_c_*^2^.

The negative TA feature below the bandgap, around 1.58 eV, has been attributed to various effects^6^,^17^, yet a clear understanding is crucial to differentiate the excitonic or free charge-carrier nature of these materials. Figure 2a shows representative spectra of a series of excitation energy-dependent pump-probe experiments, each taken at 250 fs after pump excitation, with the excitation energy gradually approaching the optical bandgap (initial, *t*=0 s, carrier densities of ∼2 × 10^17^ cm^−3^). The negative feature at 1.58 eV decreases with excitation energies approaching the bandgap, and disappears completely with resonant pumping. This observation is not in agreement with the formation of this feature from a transiently induced electro-absorption from excitonic states, which would be most pronounced under resonant pumping. In Fig. 2b, we compare the normalized carrier temperature decays (cf. Fig. 1b) with the normalized kinetics of the sub-bandgap feature. Both data sets show slower decay with increasing carrier densities (Table 1). However, the carrier cooling kinetics are consistently faster compared with the TA kinetics at 1.58 eV for each given carrier density.

We note that these trends in carrier cooling and sub-bandgap feature kinetics are also seen in CH_3_NH_3_PbI_3_ films made with a PbCl_2_ precursor^6^,^17^ (Supplementary Fig. 3), rather than the Pb(CH_3_COO)_2_ precursor (the material is sometimes written as CH_3_NH_3_PbI_3-*x*_Cl_*x*_), which illustrates that the effects can be generalized to perovskite films of different preparation routes. The difference between these measured timescales leads to the following description of the early time photophysics: immediately on photoexcitation, the presence of free carriers leads to bandgap renormalization (on timescales faster than our temporal resolution, ∼100 fs), which reduces the bandgap. Carriers are left (after rapid thermalization) in a Fermi-Dirac distribution with a high effective carrier temperature (Fig. 2c). Hot-carrier distributions occupy fewer states at the new, lowered band edge than cooler-carrier distributions. Thus, initially, the TA signal at the renormalized band edge shows a PIA caused by the newly created optical transitions due to the modified band edge states. As the carrier temperature reduces, gradual occupation of the renormalized energy states leads first to a reduction of PIA and eventually to a positive bleach signal. This interpretation is supported by established theories for inorganic semiconductors^28^.

As a test of this interpretation, we assume that the signal at 1.58 eV is proportional to the occupation of sub-bandgap states created by bandgap renormalization, which is given by the product of Fermi distribution *f*(*E*,*T*_c_) and the unchanged density of states near the band edge; therefore, Δ*T*/*T*[1.58 eV]∝*f*(*E*,*T*_c_). The change of the Fermi function with carrier temperature, ∂*f*/∂*T*_c_, can be approximated for large values of *T*_c_ (which are present at early times after excitation) as:


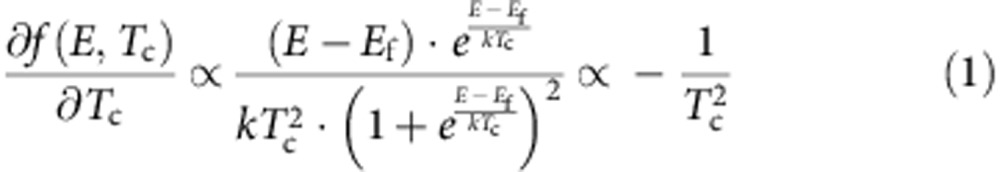


This relation can be evaluated from the measured values for (∂*T*_c_)/(∂*t*), that is, the derivative of the cooling curve (cf. Fig. 1b) and (∂*f*)/(∂*t*), which is proportional to the derivative of the 1.58 eV TA kinetics (cf. Fig. 2b). The calculated values for ∂*f*/∂*T*_c_ for different carrier densities show a linear relation against
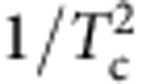
 (Fig. 2d), which matches with relation (1). We therefore attribute the sub-bandgap PIA to a TA response caused by hot-carriers cooling down to occupy the modified band edge created by ultrafast bandgap renormalization. We note that for longer time delays (>5 ps) the bandgap reduction is not simply observed as a red shift of the band edge bleach peak. We attribute this to a partial compensation of the red shift by a Burstein–Moss filling of bands by excited carriers^19^. In the following section, we address this band-filling effect in detail.

### Photoinduced reflection due to refractive index changes

Photoexcited states in all semiconducting materials induce changes in the real and imaginary parts of the dielectric function. This leads to changes in absorption (imaginary part) and reflectivity (real part), which can be substantial for materials with significant values of refractive index such as lead halide perovskites^9^ (Supplementary Fig. 4). In recent times, TA spectroscopy has been often applied to materials with low refractive indices, such as organic semiconductors, where the reflectivity change is neglible. It is, however, important to include these effects for materials with larger refractive index, where the ratio of real and imaginary part of the dielectric function determines the relative contribution of the photoinduced reflection changes. We performed simultaneous transient reflection and TA measurements on CH_3_NH_3_PbI_3_ films prepared with a PbCl_2_ precursor, denoting the transmitted and reflected component of the probe beam without pump as ‘*T*' and ‘*R*'.

**Figure 3:**
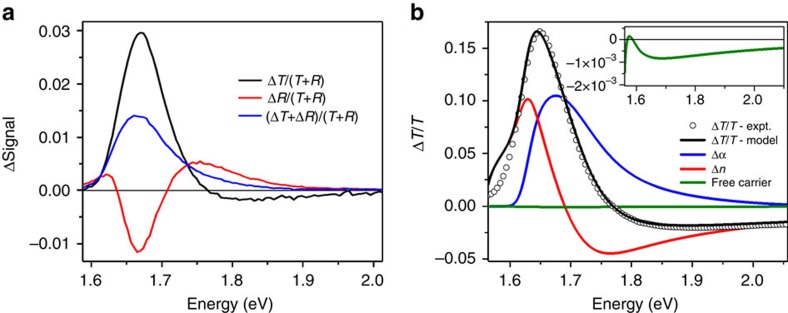
Transient absorption and reflection spectra of CH_3_NH_3_PbI_3_ films on Al_2_O_3_. (**a**) Photoinduced relative spectral change in transmitted (black line) and reflected (red line) probe intensity, weighted by the sum of transmitted (T) and reflected (R) probe intensity without photoexcitation. Spectra are taken at 5 ps after excitation (carrier density of ∼5 × 10^17^ cm^−3^) with a 2.07 eV vertically polarized pump and a p-polarized probe beam at an incident angle of *θ*=45°. The blue line is a summation of the transient transmission and reflection spectra with a common weighting (
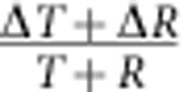
) and shows a rough approximation of the ‘intrinsic' transient absorption response accounting for changes in reflectivity. (**b**) Photoinduced change in transmitted probe signal Δ*T*/*T* of CH_3_NH_3_PbI_3_ (black circles) taken at 4 ps time delay with 2.25 eV excitation energy, initial (*t*=0) excitation density *N*=6.4 × 10^18^ cm^−3^, incident probe angle=90°, probe polarization=54.7°. The black line is a fitted spectrum according to the model presented in the text (*m*_r_=0.14 *m*_o_, Γ=0.013 eV, *a*=3, Δ*E*_BGR_=5 meV). The blue, red and green lines show the components of the modelled Δ*T*/*T* from changes in absorption, *Δα* (blue line), changes in refractive index, *Δn* (red line), and free-carrier absorption (green line, and inset). The change in refractive index gives a significant contribution to the experimental TA spectrum while the free-carrier absorption shows a negligible contribution.

Figure 3a shows the simultaneously resolved differential transmission (
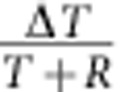
, black line) and reflection (
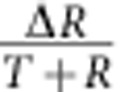
 , red line) spectra taken 6 ps after excitation at an initial (*t*=0 s) carrier density of ∼5 × 10^17^ cm^−3^ (pump 2.07 eV, pump-probe polarization angle 54.7° (‘magic angle'), incident probe angle *θ*=45°). The photoinduced reflection signal also shows a strong angular and polarization dependence (Supplementary Fig. 5). A good estimate of the ‘intrinsic' TA response can be recovered from the sum of the measured signals, 
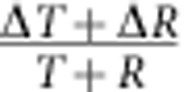
, (Fig. 3a, blue line). The difference between transient reflection and transmission spectra indicates significant photoinduced reflection differences caused by changes in refractive index. Comparing the intrinsic absorption spectrum (blue line) with the conventional differential transmission spectrum (black line), we see that reflection changes lead to a significant sharpening and enhancement of the ground-state absorption bleach, as well as the high-energy negative transmission feature. The fact that the transient reflectivity change accounts for the above bandgap negative spectral feature is significant, as this rules out the origins of this feature being due to a PIA of a higher lying state^29^, or a very large bandgap renormalization. We note that similar photoinduced reflection changes are observed in films made from acetate precursor (Supplementary Fig. 6).

The transient photoresponse is reproduced using a simple model on the basis of statefilling that takes refractive index changes into account. This enables quantification of important parameters such as the effective mass. We present here the basic parts of the model with full details in the Supplementary Methods section and Supplementary Figs 13–16. We assume a Fermi-Dirac distribution of photocarriers in parabolic bands^30^, which gives us a carrier-dependent absorption coefficient;


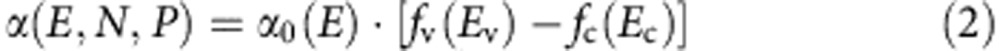


where *α*_0_(*E*) is the absorption coefficient, *N* and *P* are the concentrations of electrons and holes, *E*_v_ and *E*_c_ are the energies of the conduction and valence band onsets, and *f*_v_ and *f*_c_ are the Fermi-Dirac distribution functions. We obtain a change in absorption coefficient due to carrier injection, and subsequently a change in refractive index through Kramers–Kronig transformation^31^. We calculate the proportion of reflected light at perovskite–glass and perovskite–air interfaces from Fresnel equations^32^. For a Beer–Lambert absorption profile and a probe at normal incidence to the sample, we obtain for the relative change in transmitted probe signal:


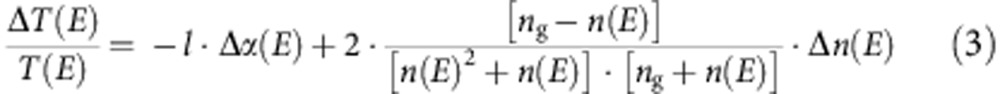


with the sample thickness *l*, the refractive index *n(E)* and the refractive index of the glass substrate, *n*_g_=1.46. As the model is symmetric with respect to the curvature of conduction and valence bands, it does not distinguish whether the electron or hole effective mass is larger. We therefore take as our key parameters the reduced effective mass, *m*_r_, and an effective mass asymmetry defined as the ratio of effective masses of the carriers in the two bands.

Figure 3b shows the change in transmitted probe signal of CH_3_NH_3_PbI_3_ (black circles) taken with normal incidence probe and initial (*t*=0) carrier density of *N=*6.4 × 10^18^ cm^−3^. The spectrum is well reproduced with a fit (black line) on the basis of the expression for Δ*T*/*T* given in the equation (3). The parameters used in this fit are *m*_r_=0.14 *m*_o_, *a*=3, *E*_g_=1.615 eV, an inhomogeneous broadening parameter, Γ=0.013 eV, and an absorption constant, *C*=0.58 m^−1^ s^−1/2^. We split the modelled spectrum into its constituent components (Fig. 3b) to illustrate the contributions due to carrier-induced changes in absorption (blue line), changes in refractive index (red line) and a much smaller free-carrier absorption (green line). The general applicability of the model is underlined by good fits to angular-dependent transient reflection and absorption data (Supplementary Fig. 7). The good agreement of the model with experimental data stresses the significance of including transient refractive index effects in the analysis of optical spectra under illumination. We note that the observed photoinduced reflectivity changes affect the fabrication of optical feedback structures and have to be taken into account in the analysis of external quantum yield spectra in photovoltaic devices.

### Effective mass calculation from reflection-corrected TA spectra

**Figure 4:**
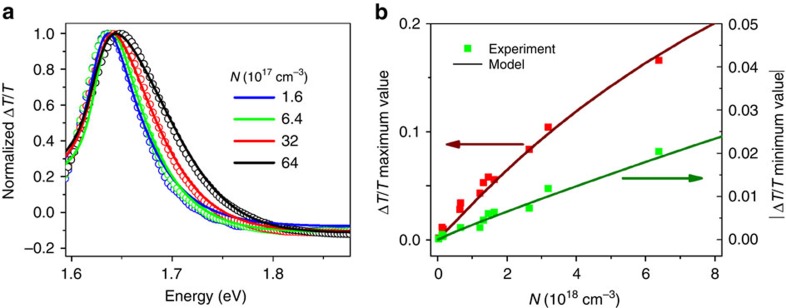
Effective mass calculation from TA spectra corrected for reflection changes. (**a**) Normalized photoinduced change in transmitted probe signal Δ*T*/*T* (open circles) for CH_3_NH_3_PbI_3_ for a range of carrier densities, taken at 4 ps time delay with 2.25 eV excitation energy, incident probe angle=90° and probe polarization=54.7°. Global fits of the experimental data to the model shown in Fig. 3b and outlined in the text are shown as solid lines. The parameters for the model are *m*_r_=0.14 *m*_o_, Γ=0.013 eV, *a*=3 and *C*=0.58 m^−1^ s^−1/2^. The model captures the Burstein−Moss spectral broadening with increasing carrier density, which is controlled by the reduced effective mass. (**b**) Carrier density dependence of the spectral maxima and minima of the photoinduced change in transmitted probe signal Δ*T*/*T*. Experimental maxima (red squares) and absolute value of spectral minima (green squares) are compared with the model shown in Fig. 3b and outlined in the text (dark red and dark green lines for maxima and minima, respectively). The model captures the saturation behaviour of the peak in transmitted probe signal Δ*T*/*T*.

While refractive index changes have little effect on extracted carrier temperatures or sub-bandgap PIA kinetics (Supplementary Fig. 8), they significantly affect estimates of the reduced effective mass. For an estimate of the effective mass including transient reflectivity effects, we perform a global fit of the model in the equation (3) to TA spectra at a number of different carrier densities (Fig. 4a). The data are normalized to highlight its most important characteristic; the spectral broadening with increasing carrier density (Burstein–Moss shift). For a bandgap of *E*_g_=1.62 eV, the global fit gives an effective mass of *m*_r_=0.14 *m*_o_ and Γ=0.013 eV, *C*=0.58 m^−1^ s^−1/2^ and *a*=3 (fitted residuals <10^−3^). The effect of individually varying these parameters, while keeping the others constant (Supplementary Fig. 9) gives us an estimate of the error in the reduced effective mass of ±0.04 *m*_o_. We stress that varying the effective mass asymmetry factor, *a*, has very little effect on the effective mass. In addition, Fig. 4b shows the carrier density dependence of the spectral maxima and minima of the experimental data and fits to the model with parameters as above, which captures the observed peak saturation behaviour for carrier densities above *N ∼*2 × 10^18^ cm^−3^. Besides the spectral broadening, the saturation behaviour is another quantity that is directly dependent only on the reduced effective mass. We note that this analysis is generally applicable to films made by other preparation routes (Supplementary Fig. 10).

Previous analysis has also focused on the spectral broadening of TA spectra (without accounting for refractive index change) to estimate the reduced effective mass^19^. Performing the same analysis on our data (without refractive index change) significantly overestimates the carrier effective mass for CH_3_NH_3_PbI_3_, at *m*_r_=0.38 *m*_o_ (Supplementary Fig. 11). The smaller value of *m*_r_=0.14 *m*_o_ reported here is more consistent with the high photovoltaic efficiencies reported in devices, as well as the value from magneto-absorption measurements^33^,^34^ and theoretical calculations^35^,^36^. TA measurements can now be reconciled with other estimates of reduced mass simply by accounting for photoinduced refractive effects in fits of TA spectra.

## Discussion

Accurate quantification of carrier effective mass and carrier cooling rates has direct implications for carrier dynamics in CH_3_NH_3_PbI_3_, and hence for its optoelectronic applications. The slow carrier cooling may be exploited to increase the efficiency of solar photovoltaic devices if hot-carrier extraction schemes can be implemented. The pronounced phonon bottleneck also suggests that hot-carrier dynamics must be accounted for in attempts to create an electrically pumped perovskite laser. The long carrier cooling timescale raises questions about the nature of carrier interactions with the lattice modes in these materials and the effect of the organic cation on the vibrational modes. These questions can be addressed in the future by directly probing optical resonances at phonon energies and by comparing carrier cooling dynamics in perovskites of different compositions.

The carrier effective mass measured for CH_3_NH_3_PbI_3_ allows us to estimate quasi-Fermi energies and thereby predict the carrier density at which optical gain will occur. This arises when the difference in Fermi levels becomes greater than the bandgap, and is predicted from global fits of the model presented in Fig. 4 to take place at *N* ∼2.5 × 10^18^ cm^−3^ (Supplementary Fig. 12). This critical density matches well with experimental reports of optical gain threshold^6^,^7^, and further supports the conclusion^6^,^9^,^17^,^19^ that for this material at room temperature, excitons play a very small role.

Fits to our experimental TA spectra are consistently better when a degree of effective mass asymmetry is included in the model. Effective mass asymmetry between electrons and holes affects carrier recombination dynamics by changing the available number of states through which recombination can occur^37^. This asymmetry might be of importance to photovoltaic devices and can partially explain the surprisingly slow recombination rates in these materials^6^,^19^,^38^. Currently, there is still some uncertainty as to whether electrons or holes have the higher band curvature^20^,^36^,^39^,^40^,^41^.

In summary, we resolved hot-carrier cooling dynamics in CH_3_NH_3_PbI_3_ for the first time. In spite of the disordered nature of these polycrystalline materials, slow cooling dynamics and a phonon bottleneck effect were resolved. These effects are consistent with excellent transport properties and must be considered in the design of optical gain devices. Bandgap renormalization operates across a wide range of carrier densities and, together with hot-carrier cooling, explains the short-lived TA feature below the optical band edge. The effects of bandgap renormalization and the Burstein–Moss effect on the band edge partially compensate each other, leading to only a small shift in the observed optical band edge. TA spectra also feature a significant contribution from photoinduced reflectivity changes, which must be accounted for when fitting spectra to a simple band-filling model. In doing so, we accurately determine a significantly smaller value of the reduced effective mass, *m*_r_=0.14 *m*_o_, than previous estimates that do not consider reflectivity effects. The small reduced effective mass is consistent with high charge mobilities and low optical gain thresholds, and thus goes well towards explaining the remarkable semiconductor properties of hybrid lead halide perovskites. These insights into the intrinsic photophysical parameters of CH_3_NH_3_PbI_3_, and the methods developed to access them, will help to guide the development of future photovoltaic devices, light-emitting diodes and electrically pumped lasers made from this family of materials.

## Methods

### Film preparation

Methylammonium iodide CH_3_NH_3_I synthesis is described elsewhere^1^,^25^,^29^. For the lead acetate (Pb(CH_3_COO)_2_)-based perovskite thin film fabrication: 3:1 molar stoichiometric ratios of CH_3_NH_3_I and Pb(CH_3_COO)_2_ (Sigma Aldrich 99.999% pure) were made in *N*,*N*-dimethylformamide (DMF) in 20 wt% concentration. These solutions were spun inside a nitrogen filled glove box on quartz substrates at 2,000 r.p.m. for 60 s followed by 3 min of thermal annealing at 100 °C in air to form thin films. CH_3_NH_3_PbI_3_ with PbCl_2_ precusor (CH_3_NH_3_PbI_3-x_Cl_x_) samples for simultaneous transmission/reflection measurements were prepared on an Al_2_O_3_ scaffold as described here^17^ and sealed in an N_2_ cell in a glove box to minimize degradation.

### Ultrafast transient absorption

A Ti:Sapphire amplifier system (Spectra-Physics Solstice) operating at 1 KHz generated 90-fs pulses was split to given the pump and probe beam arms. The broad band probe beam was generated in a home-built noncollinear optical parametric amplifier. The pulsed excitation was provided by a TOPAS optical parametric amplifier (Light Conversion), to generate narrowband (10 nm full-width at half-maximum) pump pulses. The transmitted pulses were collected with an InGaAs dual-line array detector (Hamamatsu G11608-512) driven and read out by a custom-built board from Stresing Entwicklungsbüro.

### Simultaneous transient transmission and reflection

These measurements used the ultrafast TA spectroscopy setup described previously^42^. The key difference in this case was that the two channels of the linear photodiode array were used for simultaneous measurement of transmitted and reflected probe beams on a shot-to-shot basis. The two probe beams were coupled into the spectrometer via optical fibres to ensure that efficient coupling of both beams enabled correct weighting of T and R channels. Measurements were carried out with pump and probe pulses incident through the substrate side, owing to its smooth interface with the perovskite layer. Reflections from glass–air interfaces were corrected for using the Fresnel equations.

### Steady-state absorption measurements

Reflection-corrected ultraviolet–visible absorption spectra were recorded using a PerkinElmer Lambda 750 spectrophotometer. For the photothermal deflection spectroscopy, a monochromatic pump beam was shined on to the sample (film on Quartz substrate), which on absorption produces a thermal gradient near the sample surface via non-radiative relaxation-induced heating. This results in a refractive index gradient in the area surrounding the front of the sample surface. This refractive index gradient is further enhanced by immersing the sample in an inert liquid FC-72 Fluorinert (3M Company), which has a high refractive index change per unit change in temperature. A fixed wavelength CW probe laser beam is passed through this refractive index gradient producing a deflection, which is proportional to the light absorbed in the sample at that particular wavelength, which is further detected by a photodiode and lock-in amplifier combination.

### Ellipsometry

Variable angle spectroscopic ellipsometry was measured using a M-2000 ellipsometer (Woollam Co.) with rotating compensator in the wavelength range from 400 to 800 nm on samples prepared on Si(100) with a native oxide layer. The data were fitted using a layered optical model, taking the optical uniaxial anisotropy of the samples into account and based on a film thickness determined by X-ray reflectivity measurements of the same samples.

## Additional information

**How to cite this article:** Price, M. B. *et al.* Hot-carrier cooling and photoinduced refractive index changes in organic–inorganic lead halide perovskites. *Nat. Commun.* 6:8420 doi: 10.1038/ncomms9420 (2015).

## Supplementary Material

Supplementary Information Supplementary Figures 1-16, Supplementary Methods and Supplementary References

## Figures and Tables

**Table 1:** Fluence dependence of the carrier cooling times *τ*_T_C__ and transient absorption decay times *τ*_Δ*T*/*T*_ at 1.58 eV.

***N*** **(10**^17^ **cm**^−3^)	**1.6**	**6.4**	**32**	**64**
*τ*_T_C__ (fs)	210	230	340	770
*τ*_Δ*T*/*T*_ (fs)	430	450	500	600

## References

[b1] Lee M. M., Teuscher J., Miyasaka T., Murakami T. N. & Snaith H. J. Efficient hybrid solar cells based on meso-superstructured organometal halide perovskites. Science 338, 643–647 (2012) . 23042296 10.1126/science.1228604

[b2] Kim H.-S. *et al.* Lead iodide perovskite sensitized all-solid-state submicron thin film mesoscopic solar cell with efficiency exceeding 9%. Sci. Rep. 2, 591 (2012) . 22912919 10.1038/srep00591 PMC3423636

[b3] Jeon N. J. *et al.* Compositional engineering of perovskite materials for high-performance solar cells. Nature 517, 476–480 (2015) . 25561177 10.1038/nature14133

[b4] Zhou H. *et al.* Interface engineering of highly efficient perovskite solar cells. Science 345, 542–546 (2014) . 25082698 10.1126/science.1254050

[b5] Tan Z.-K. *et al.* Bright light-emitting diodes based on organometal halide perovskite. Nat. Nanotechnol. 9, 687–692 (2014) . 25086602 10.1038/nnano.2014.149

[b6] Deschler F. *et al.* High photoluminescence efficiency and optically-pumped lasing in solution-processed mixed halide perovskite semiconductors. J. Phys. Chem. Lett. 5, 1421 (2014) . 26269988 10.1021/jz5005285

[b7] Xing G. *et al.* Low-temperature solution-processed wavelength-tunable perovskites for lasing. Nat. Mater. 13, 476–480 (2014) . 24633346 10.1038/nmat3911

[b8] Zhang Q., Ha S. T., Liu X., Sum T. C. & Xiong Q. Room-temperature near-infrared high-Q perovskite whispering-gallery planar nanolasers. Nano Lett. 14, 5995–6001 (2014) . 25118830 10.1021/nl503057g

[b9] Lin Q. *et al.* Electro-optics of perovskite solar cells. Nat. Photon. 9, 106–112 (2014) .

[b10] Ross R. T. Efficiency of hot-carrier solar energy converters. J. Appl. Phys. 53, 3813 (1982) .

[b11] Mücke O. D. & Wegener M. Hot carrier induced picosecond dynamics of a vertical cavity surface emitting laser: Influence of transverse effects. Appl. Phys. Lett. 73, 569 (1998) .

[b12] Elsässer M. *et al.* Subpicosecond switch-off and switch-on of a semiconductor laser due to transient hot carrier effects Subpicosecond switch-off and switch-on of a semiconductor laser due to transient hot carrier effects. Appl. Phys. Lett. 853, 1–4 (2013) .

[b13] Pavan P., Bez R., Olivo P. & Zanoni E. Flash memory cells-an overview. Proc. IEEE 85, 1248–1271 (1997) .

[b14] Shah J. & Leite R. C. C. Radiative recombination from photoexcited hot carriers in GaAs. Phys. Rev. Lett. 22, 22–25 (1969) .

[b15] Shah J. Hot electrons and phonons under high intensity photoexcitation of semiconductors. Solid State Electron. 21, 43–50 (1978) .

[b16] Nuss M. C., Auston D. & Capasso F. Direct subpicosecond measurement of carrier mobility of photoexcited electrons. Phys. Rev. Lett. 58, 2355–2358 (1987) . 10034724 10.1103/PhysRevLett.58.2355

[b17] Chen K., Barker A. J., Morgan F. L. C., Halpert J. E. & Hodgkiss J. M. Effect of carrier thermalization dynamics on light emission and amplification in organometal halide perovskites. J. Phys. Chem. Lett. 6, 153 (2015) . 26263104 10.1021/jz502528c

[b18] Xiao Z. *et al.* Giant switchable photovoltaic effect in organometal trihalide perovskite devices. Nat. Mater. 14, 1–7 (2014) . 10.1038/nmat4150 25485985

[b19] Manser J. S. & Kamat P. V. Band filling with free charge carriers in organometal halide perovskites. Nat. Photon. 8, 737–743 (2014) .

[b20] Stranks S. D. *et al.* Electron-hole diffusion lengths exceeding 1 micrometer in an organometal trihalide perovskite absorber. Science 342, 341–344 (2013) . 24136964 10.1126/science.1243982

[b21] Xing G. *et al.* Long-range balanced electron- and hole-transport lengths in organic-inorganic CH3NH3PbI3. Science 342, 344–347 (2013) . 24136965 10.1126/science.1243167

[b22] Zhang W. *et al.* Ultra-smooth organic-inorganic perovskite thin-film formation and crystallization for Efficient Planar Heterojunction Solar Cells. Nat. Commun. 6, 6142 (2014) . 25635571 10.1038/ncomms7142

[b23] Shank C. V., Fork R. L., Leheny R. F. & Shah J. Dynamics of photoexcited GaAs band-edge absorption with subpicosecond resolution. Phys. Rev. Lett. 42, 112–115 (1979) .

[b24] Shah J. Photoexcited hot carriers: From cw to 6 fs in 20 Years. Solid State Electron. 32, 1051–1056 (1989) .

[b25] Rosenwaks Y. *et al.* Hot-carrier cooling in GaAs: quantum wells versus bulk. Phys. Rev. B 48, 6–9 (1993) . 10.1103/physrevb.48.14675 10007896

[b26] Brivio F. *et al.* Lattice dynamics and vibrational spectra of the orthorhombic, tetragonal and cubic phases of methylammonium lead iodide. Preprint at <http://arXiv:1504.07508> (2015) .

[b27] Skolnick M. S. & Bimberg D. Angular-dependent magnetoluminescence study of the layer compound 2H-PbI2. Phys. Rev. B 18, 7080 (1978) .

[b28] Shah J. Ultrafast Spectroscopy of Semiconductors and Semiconductor Nanostructures Springer (1999) .

[b29] Stamplecoskie K. G., Manser J. S. & Kamat P. V. Dual nature of the excited state in organic-inorganic lead halide perovskites. Energy Environ. Sci. 8, 208–215 (2014) .

[b30] S. M. Trevor, J. B. Geoffrey & E. Brian Semiconductor Opto-Electronics Butterworths (1973) .

[b31] Stern F. Dispersion of the Index of Refraction Near the Absorption Edge of Semiconductors. Phys. Rev. 133, 1653 (1957) .

[b32] Hecht E. Optics Addison Wesley (1987) .

[b33] Tanaka K. *et al.* Comparative study on the excitons in lead-halide-based perovskite-type crystals CH3NH3PbBr3 CH3NH3PbI3. Solid State Commun. 127, 619–623 (2003) .

[b34] Miyata A. *et al.* Direct measurement of the exciton binding energy and effective masses for charge carriers in organic-inorganic tri-halide perovskites. Nat. Phys. 11, 582–587 (2015) .

[b35] Brivio F., Butler K. T., Walsh A. & van Schilfgaarde M. Relativistic quasiparticle self-consistent electronic structure of hybrid halide perovskite photovoltaic absorbers. Phys. Rev. B 89, 155204 (2014) .

[b36] Umari P., Mosconi E. & De Angelis F. Relativistic GW calculations on CH3NH3PbI3 and CH3NH3SnI3 perovskites for solar cell applications. Sci. Rep. 4, 4467 (2014) . 24667758 10.1038/srep04467 PMC5394751

[b37] Yablonovitch E. & Kane E. Reduction of lasing threshold current density by the lowering of valence band effective mass. J. Light. Technol. 4, 504–506 (1986) .

[b38] Wehrenfennig C., Eperon G. E., Johnston M. B., Snaith H. J. & Herz L. M. High charge carrier mobilities and lifetimes in organolead trihalide perovskites. Adv. Mater. 26, 1584–1589 (2014) . 24757716 10.1002/adma.201305172 PMC4722848

[b39] Menéndez-Proupin E., Palacios P., Wahnón P. & Conesa J. C. Self-consistent relativistic band structure of the CH3NH3PbI3 perovskite. Phys. Rev. B 90, 045207 (2014) .

[b40] Filippetti A. & Mattoni A Hybrid perovskites for photovoltaics: Insights from first principles. Phys. Rev. B 89, 125203 (2014) .

[b41] Edri E. *et al.* Why lead methylammonium tri-iodide perovskite-based solar cells require a mesoporous electron transporting scaffold (but not necessarily a hole conductor). Nano Lett. 14, 1000–1004 (2014) . 24475878 10.1021/nl404454h

[b42] Barker A. J., Chen K. & Hodgkiss J. M. Distance distributions of photogenerated charge pairs in organic photovoltaic cells. J. Am. Chem. Soc. 136, 12018–12026 (2014) . 25102389 10.1021/ja505380j

