# Supplementary material for: Hot-carrier cooling and photoinduced refractive index changes in organic–inorganic lead halide perovskites
Source: Nat Commun. 2015 Sep 25;6:8420. doi: 10.1038/ncomms9420 (PMC4598728; doi:10.1038/ncomms9420)
Supplement: Supplementary Information — Supplementary Figures 1-16, Supplementary Methods and Supplementary References [file ncomms9420-s1.pdf]

Supplementary Information: Hot Carrier Cooling and Photoinduced Refractive Index Changes in Organic-Inorganic Lead Halide Perovskites.

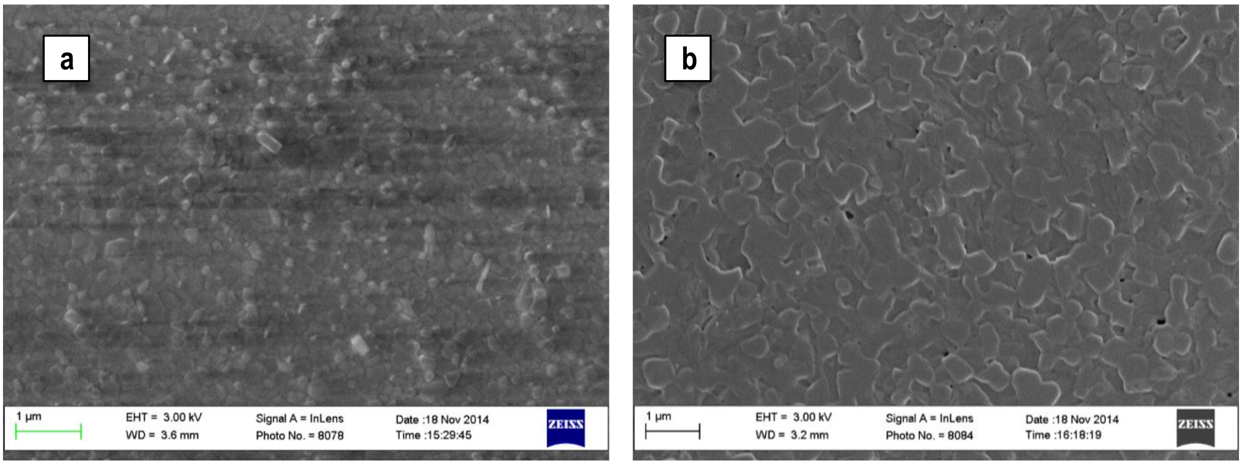

Supplementary Figure 1: Scanning electron microscopy of films of $CH_3NH_3PbI_3$ made with $Pb(CH_3COO)_2$ (a) and $CH_3NH_3PbI_3$ made with $PbCl_2$ (b)

The film of $CH_3NH_3PbI_3$ made with $Pb(CH_3COO)_2$ precursor shows crystallites of approx. 100 nm dimensions compared to the film of of $CH_3NH_3PbI_3$ made with $PbCl_2$ precursor with crystals of approximately 500-1000 nm. The smaller crystal size of the $Pb(CH_3COO)_2$ films leads to less light scatter, enabling resonant pump probe experiments and absorption measurements to be more accurate.

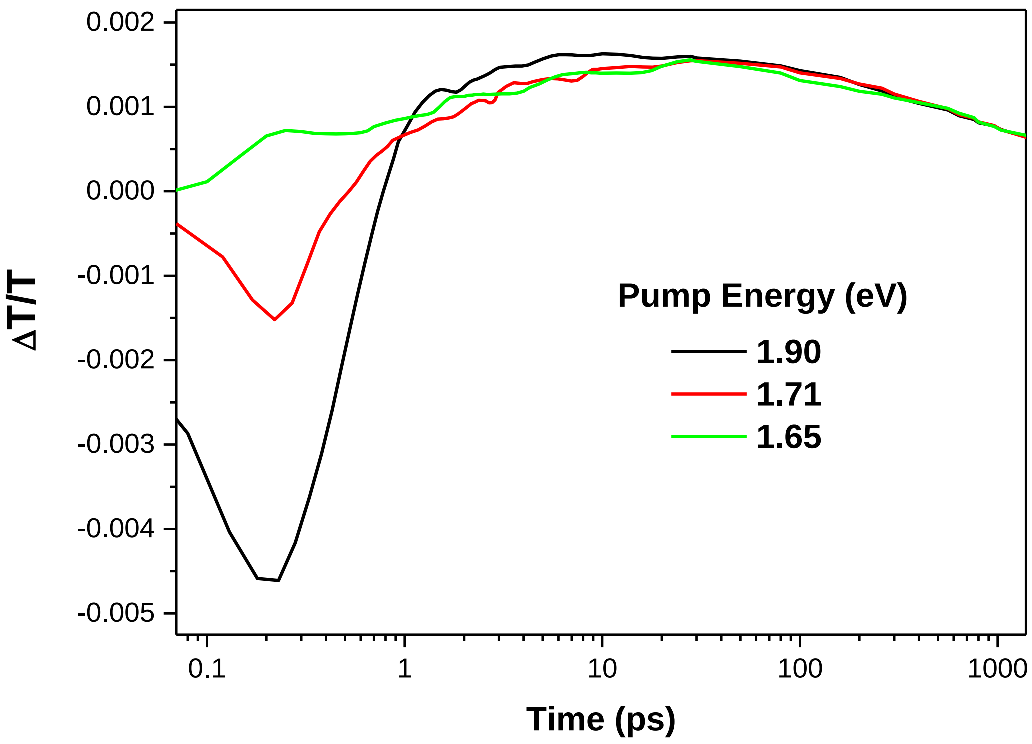

Supplementary Figure 2: Cooling rate differences for different excitation energies.

Kinetic traces of the 1.58 eV negative feature of transient absorption spectra (as proxy for carrier cooling rate) at a range of pump energies, with fluences corresponding to a carrier density of approximately $N = 2\text{x}10^{17}$ cm^{-3}. The kinetic traces of the 1.58 eV feature illustrate the bi-exponential nature of its rise time, and the disappearance of the fast rise component when pumped close to resonance.

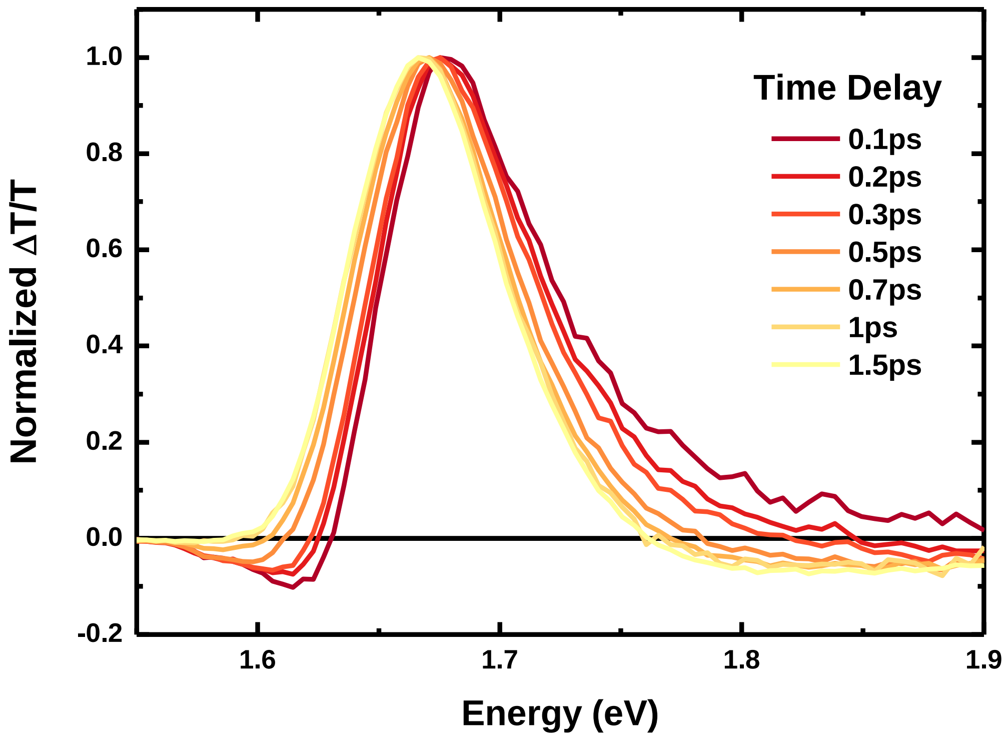

Supplementary Figure 3a: Early time TA spectra of $CH_3NH_3PbI_3$ made via the $PbCl_2$ precursor route. Films were excited at 2.07 eV with 100 fs pulses and measured at an excitation fluence of 8μJ cm^{-2}.

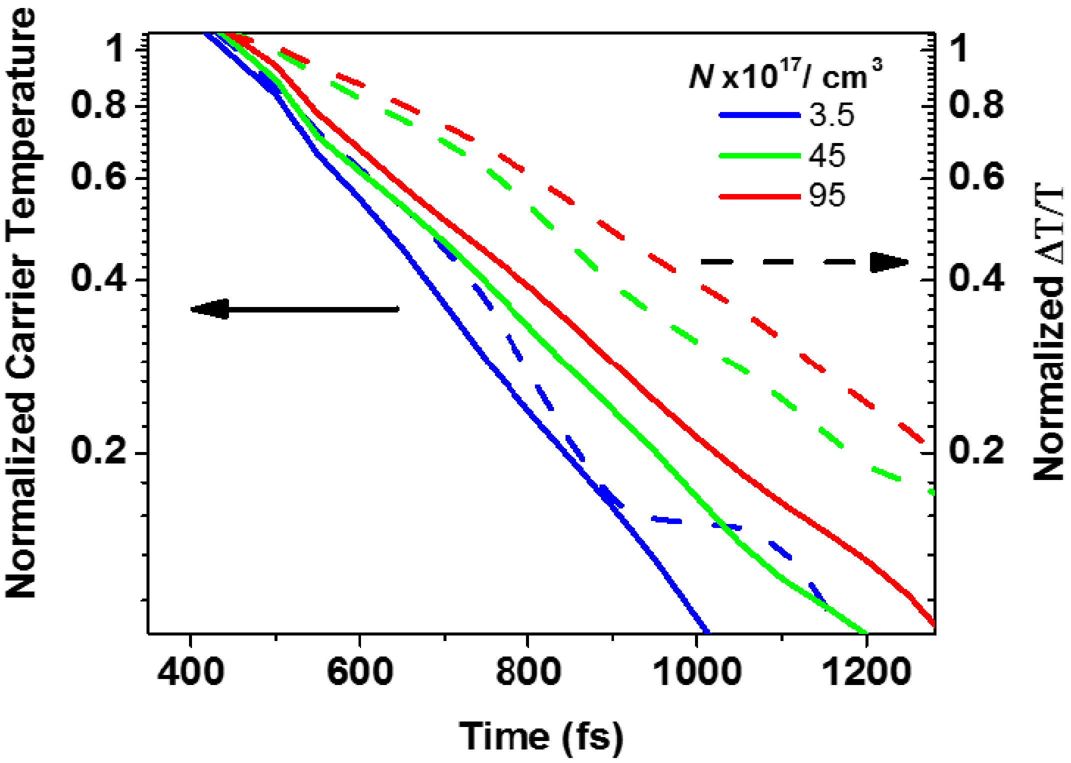

Supplementary Figure 3b: Carrier cooling and sub-bandgap TA feature of films of $CH_3NH_3PbI_3$ made with $PbCl_2$ precursor.

This figure shows the same data as that presented in the main text Figure 2b but for a sample of $CH_3NH_3PbI_3$ made with $PbCl_2$ precursor rather than $Pb(CH_3COO)_2$. It illustrates that the same trends can be seen in the material processed by a range of methods. Both, the carrier cooling and the subgap negative TA feature, show slowed rates with increasing carrier densities, and the TA feature has a consistently slower decay than the carrier temperature decay. It also shows that at similar low carrier densities i.e. N~3.5 $x10^{17}$ cm^{-3}, $PbCl_2$ precursor film has a nearly identical carrier cooling rate, of $\tau_{T_C} = 240$ fs, as the $Pb(CH_3COO)_2$ precursor films.

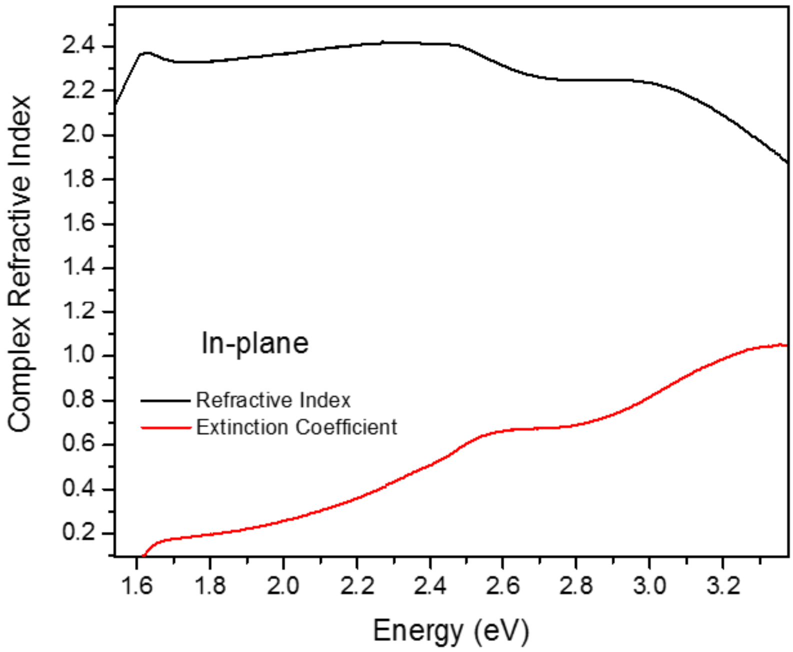

Supplementary Figure 4a

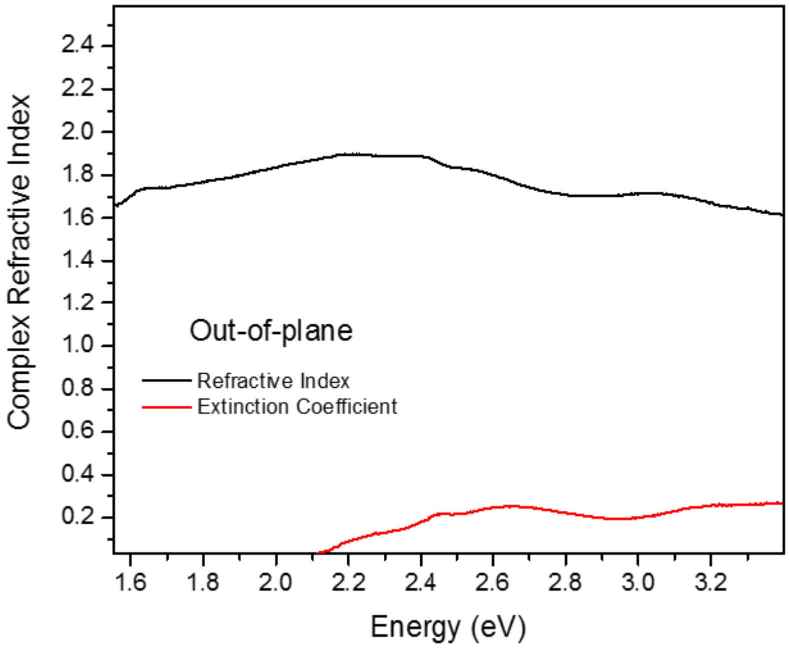

Supplementary Figure 4b

Supplementary Figure 4 Complex refractive index of $CH_3NH_3PbI_3$.

(a) In-plane component of complex refractive index of $CH_3NH_3PbI_3$. **(b)** Out-of-plane component of complex refractive index of $CH_3NH_3PbI_3$. Ellipsometric measurement as described in methods section of main text. The optical anisotropy is taken into account by a uniaxial anisotropic layer which is included in the VASE analysis software from J. Woollam Co. as the last layer of the model.

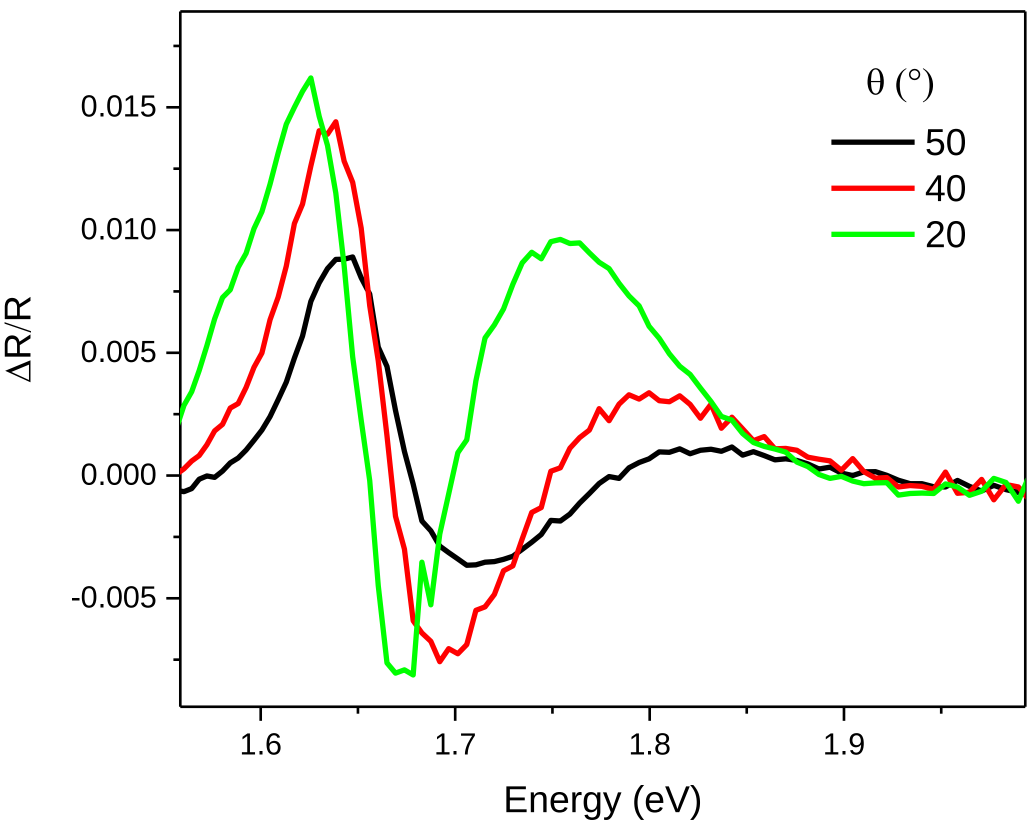

Supplementary Figure 5a

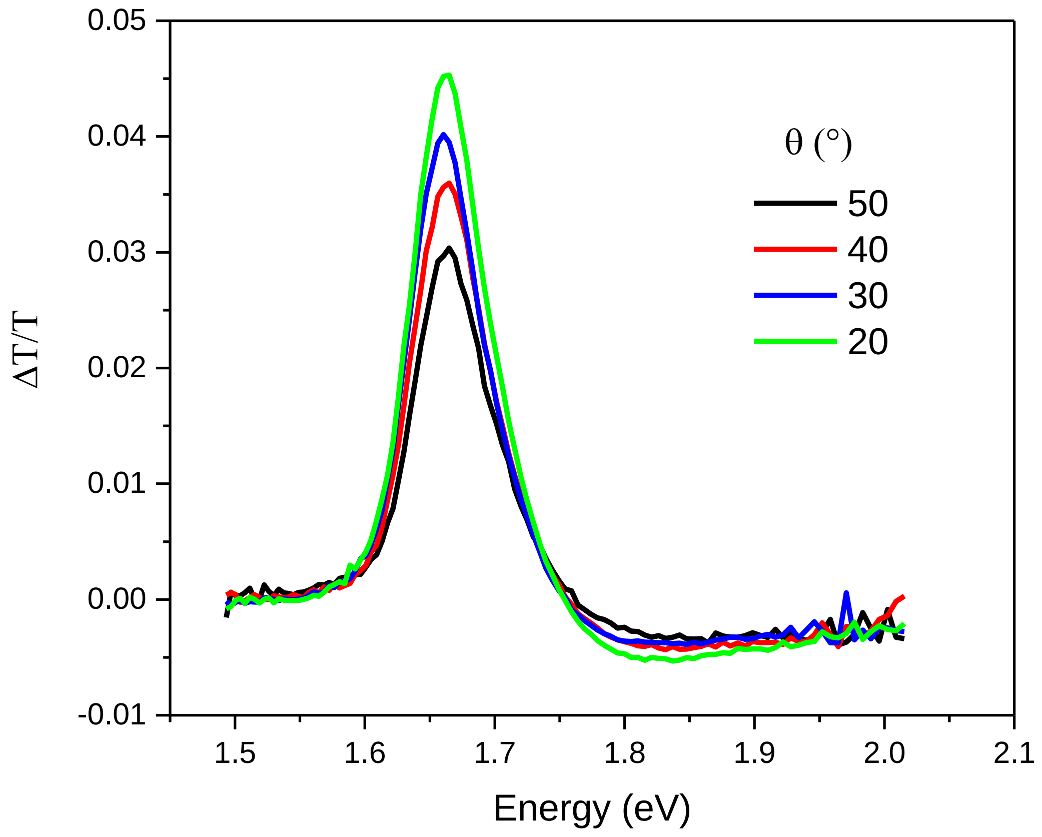

Supplementary Figure 5b

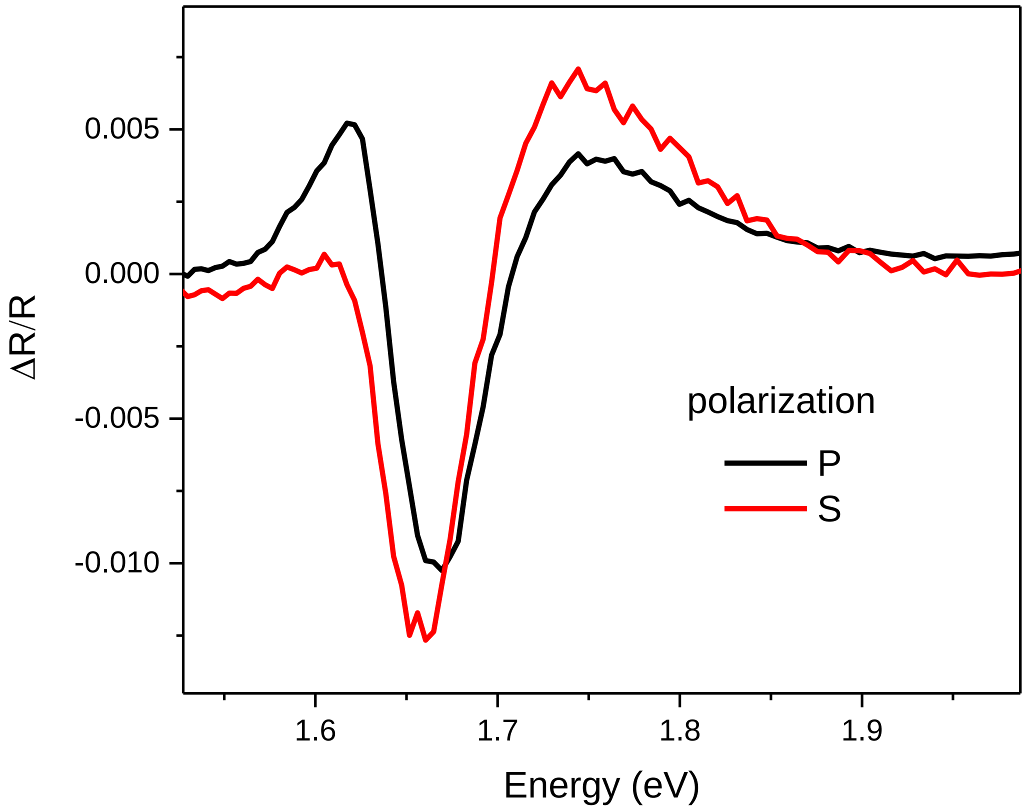

Supplementary Figure 5c

Supplementary Figure 5: Polarization and angle dependence of transient absorption and reflection

(a) Angle dependence of ΔR/R spectra of $CH_3NH_3PbI_3$ on Al_2O_3. N = 5x10^{17} cm^{-3}, taken at 5 ps after excitation with a 2.07 eV vertically polarized pump and s-polarized probe. Incident angles are as stated in legend. **(b)** Angle dependence of ΔT/T spectra of $CH_3NH_3PbI_{3-x}Cl_x$ on Al_2O_3 corresponding to the reflections measured in Supplementary Figure 5a. (**c)** Probe polarization dependence of ΔR/R spectra of $CH_3NH_3PbI_3$ on Al_2O_3, N = 5x10^{17} cm^{-3}, taken at 5 ps after excitation with a 2.07 eV vertically polarized pump.

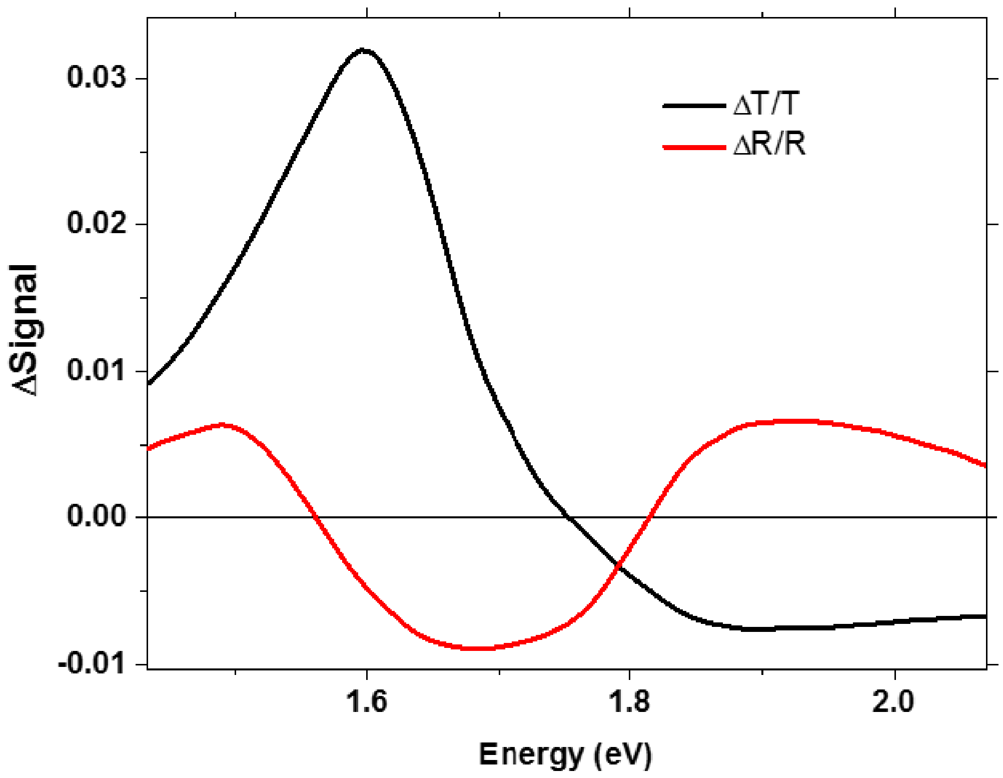

Supplementary Figure 6: Photo-induced relative spectral change in transmitted (black line) and reflected (red line) probe intensity for $CH_3NH_3PbI_3$ films on glass, fabricated from $Pb(CH_3COO)_2$ precursor. Spectra are taken at 5 ps after excitation with laser pulses (50 fs, 1 kHz) at 2.3 eV (carrier density of ~$1x10^{17}$ cm^{-3}) and probed with a broadband white-light continuum. The response shows similar photoinduced transmission and reflection changes in shape and magnitude compared to the one observed for films made from $PbCl_2$ precursor in Figure 3a.

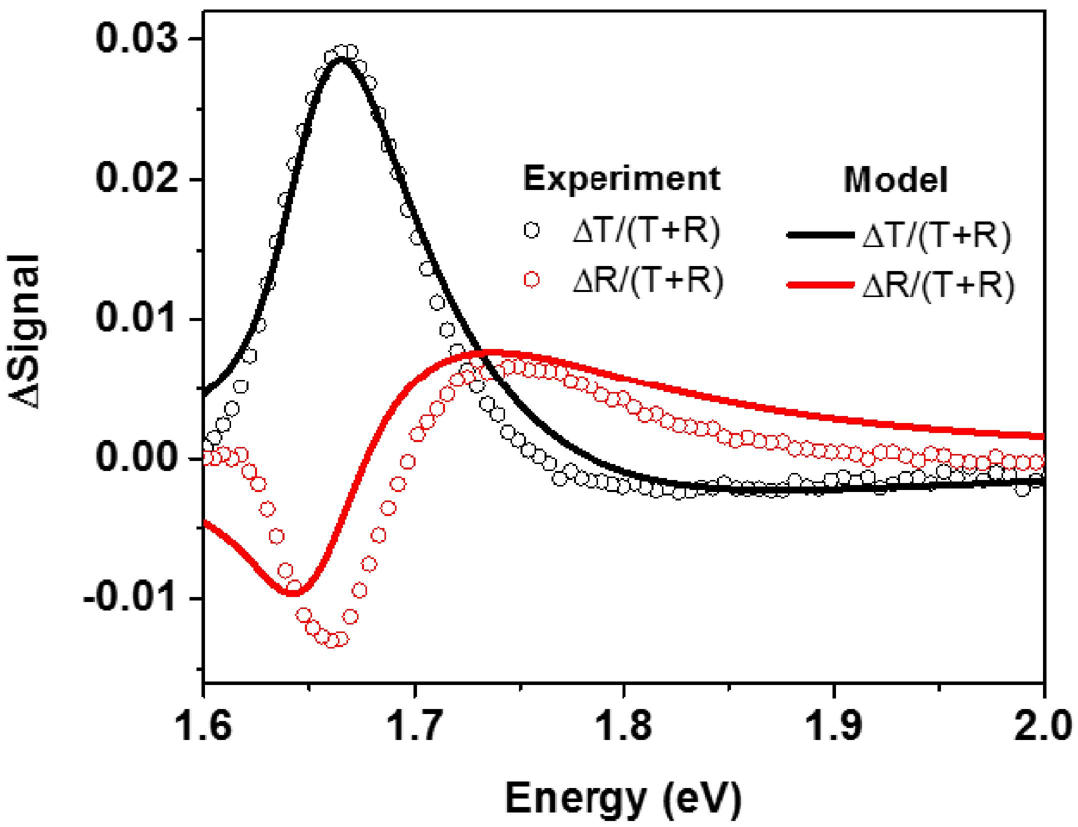

Supplementary Figure 7: Experimental and modelled transient absorption and reflection spectra of $CH_3NH_3PbI_3$on Al_2O_3

Simultaneously measured transient transmission spectrum (black circles) and transient reflection spectrum (red circles) of the sample at a carrier density of ~$5x10^{17}$ cm^{-3}, taken at 5 ps after excitation with a 2.07 eV vertically polarized pump and a p-polarized probe beam at an incident angle of θ = 45° to the sample. The solid lines are a fit to the experimental data using the simple band-filling model and reflections calculated from the angular dependent Fresnel equations. Fit parameters: E_g = 1.64 eV, m_r = 0.15 m_o, Γ = 0.03 eV, a = 1.5.

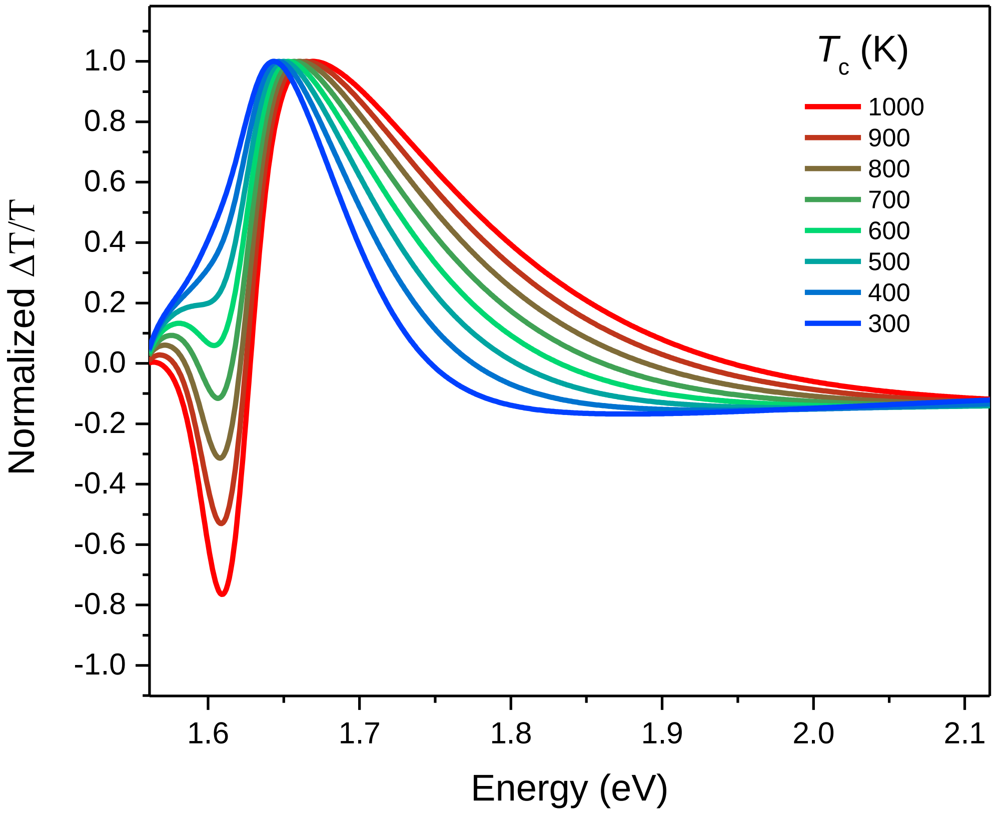

Supplementary Figure 8a

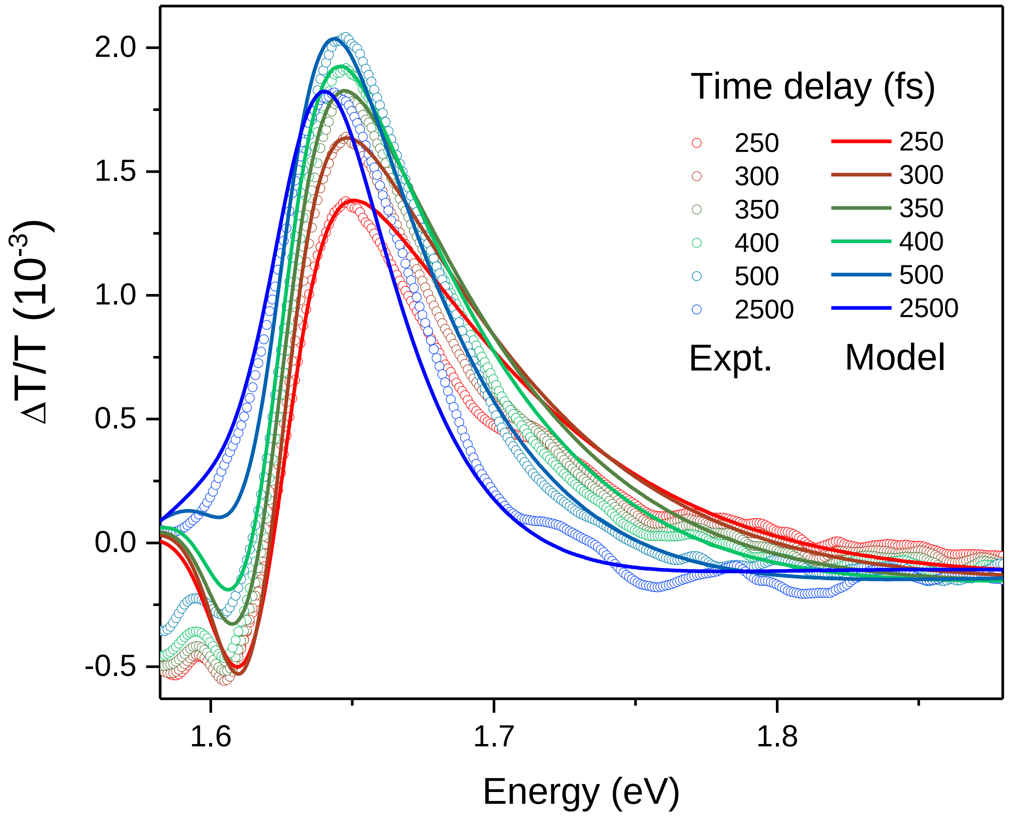

Supplementary Figure 8b

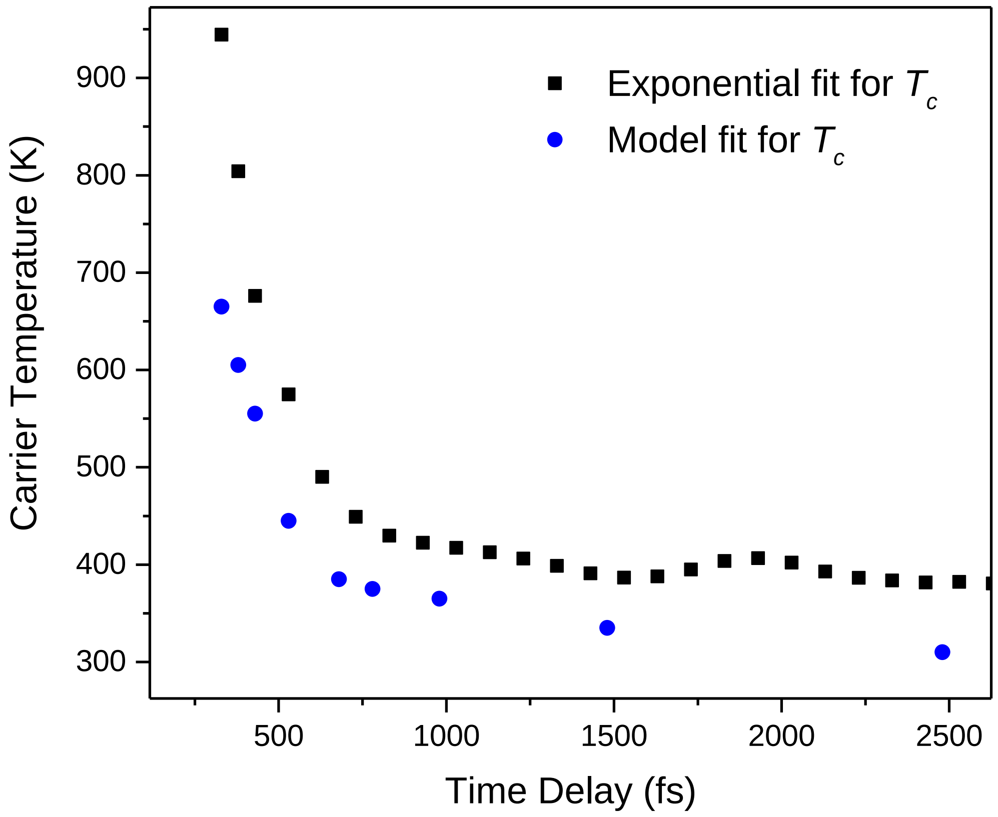

Supplementary Figure 8c

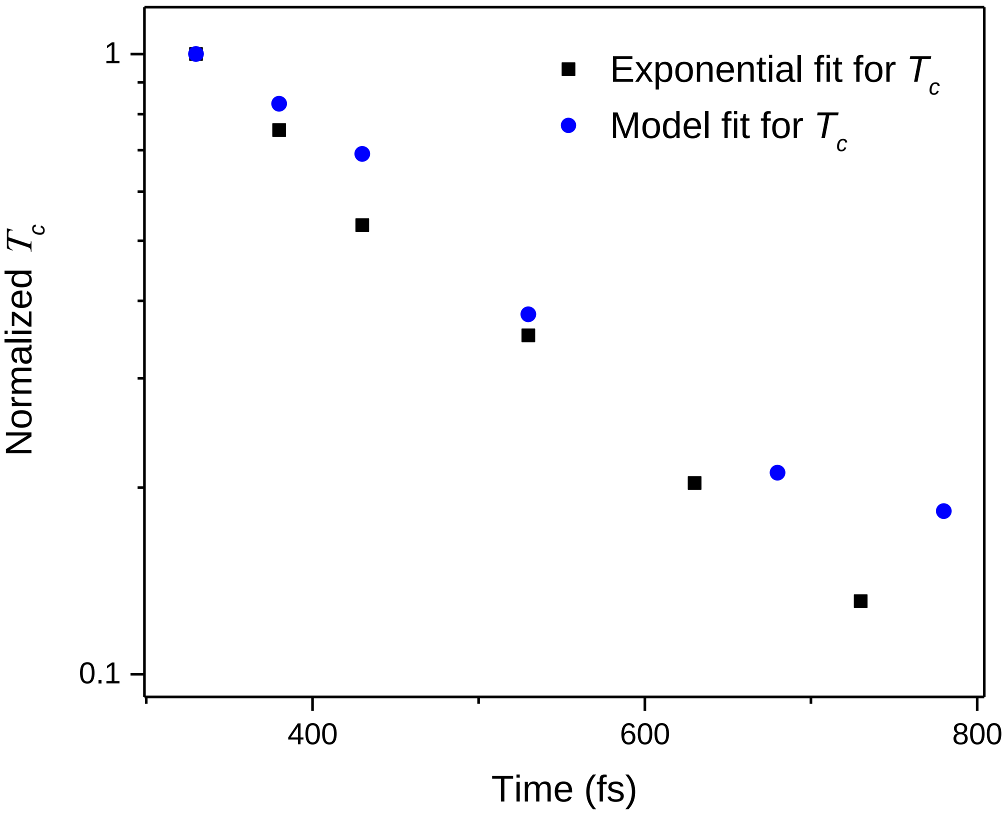

Supplementary Figure 8d

Supplementary Figure 8: Effect of refractive index changes on modelled carrier temperatures. **(a)** Model parameters; N = 6.4x10^{18} cm^{-3} , m_r = 0.14 m_o, Γ = 0.013 eV, C = 0.58 m^{-1}s$^{-1/2}$, ΔE_{BGR} = 15 meV. Temperatures stated in legend. The figure shows how the model recreates the sub-band-gap TA feature and spectral broadening for high carrier temperatures and a moderate bandgap renormalization. **(b)** Early time TA spectra of $CH_3NH_3PbI_3$ with over-layed model TA spectra. Experimental spectra obtained with 2.25 eV pump, N =1.3x10^{17} cm^{-3}. The model fits were obtained by applying the fit described in Fig 4 (parameters; m_r = 0.14 m_o, Γ = 0.013 eV, a = 3, C = 0.58 m^{-1}s$^{-1/2}$, with ΔE_{BGR} = 5 meV) assuming T_c = 300 K to the fully cooled spectra at 2500 fs. Then for the earlier, hotter spectra, the model was fitted to experiment

allowing only T_c and C as free parameters. **(c)** Carrier cooling curves of $CH_3NH_3PbI_3$, for the data described in Supplementary Figure 8b. The blue circles show the carrier temperatures obtained by the method and fits shown in Supplementary Figure 8b. The black squares show carrier temperatures obtained by the method described in Fig 1. The cooling curves show a very similar trend, differing only by a small constant temperature offset. **(d)** The data of Fig. Supplementary Figure 8c normalized and presented on a vertical log scale plot. The overlapping temperature decays show that the two methods for obtaining carrier cooling give very similar cooling rates, and thus that inclusion of refractive index changes does not change the results of the hot-carrier analysis performed in Figures 1 and 2 of the main text.

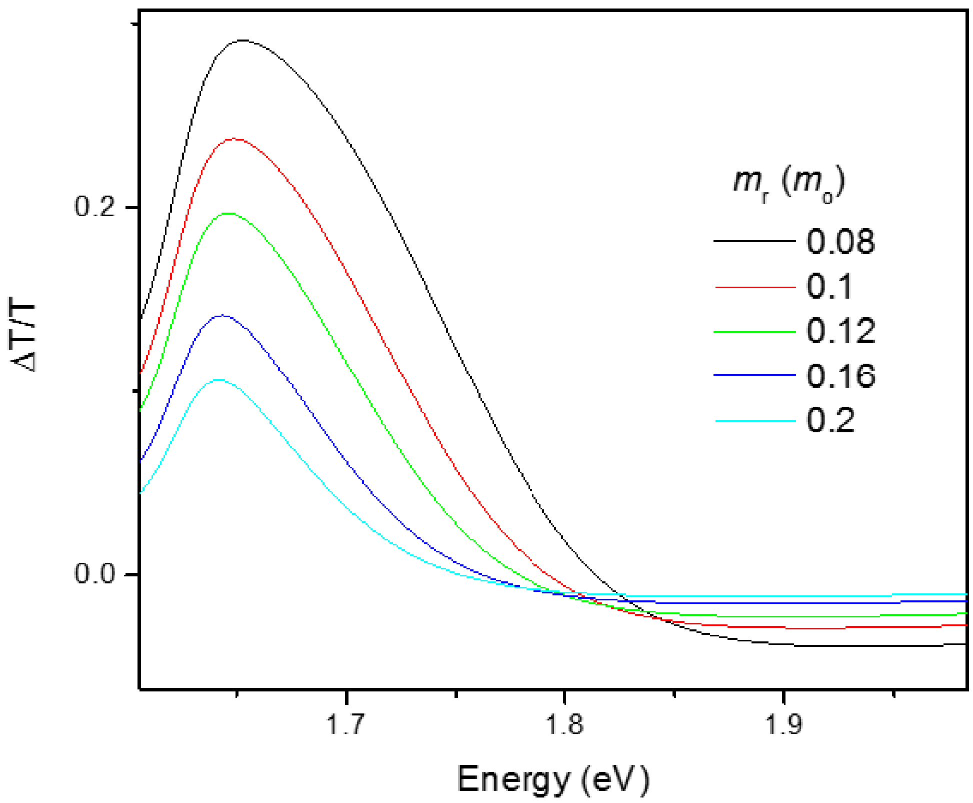

Supplementary Figure 9a

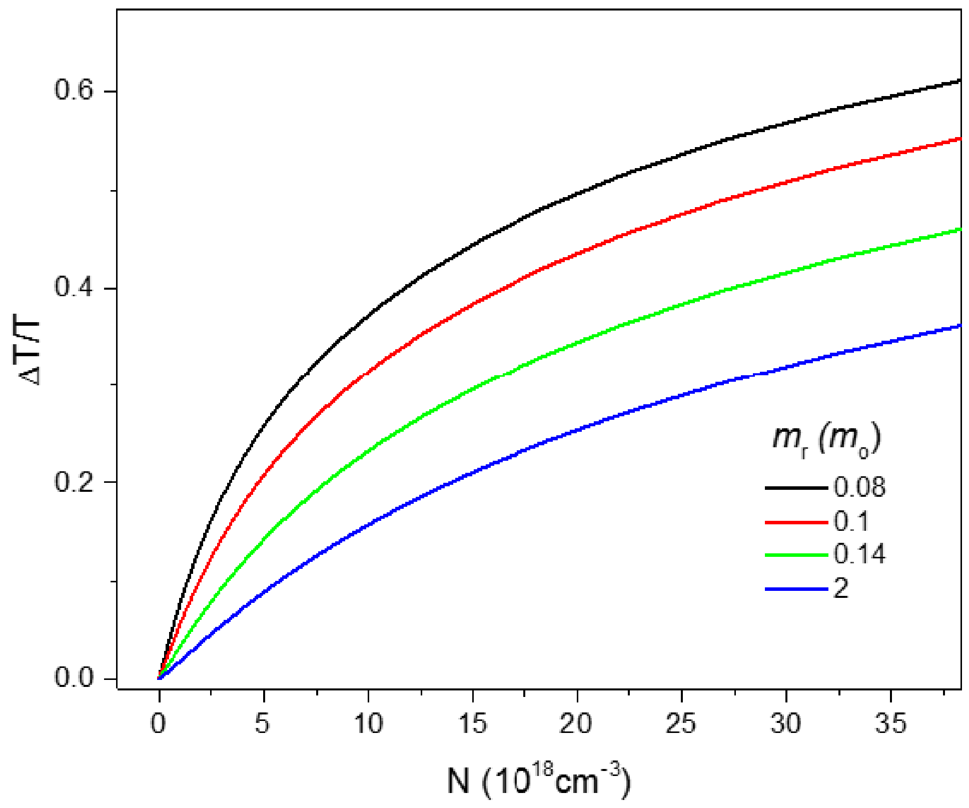

Supplementary Figure 9b

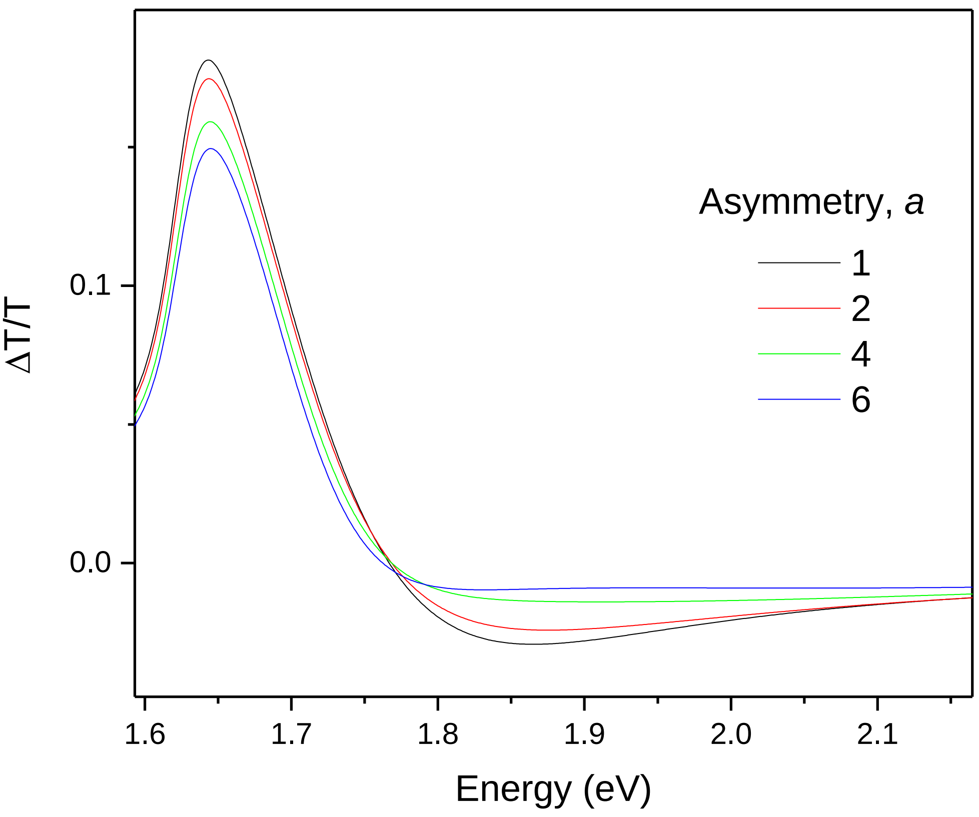

Supplementary Figure 9c

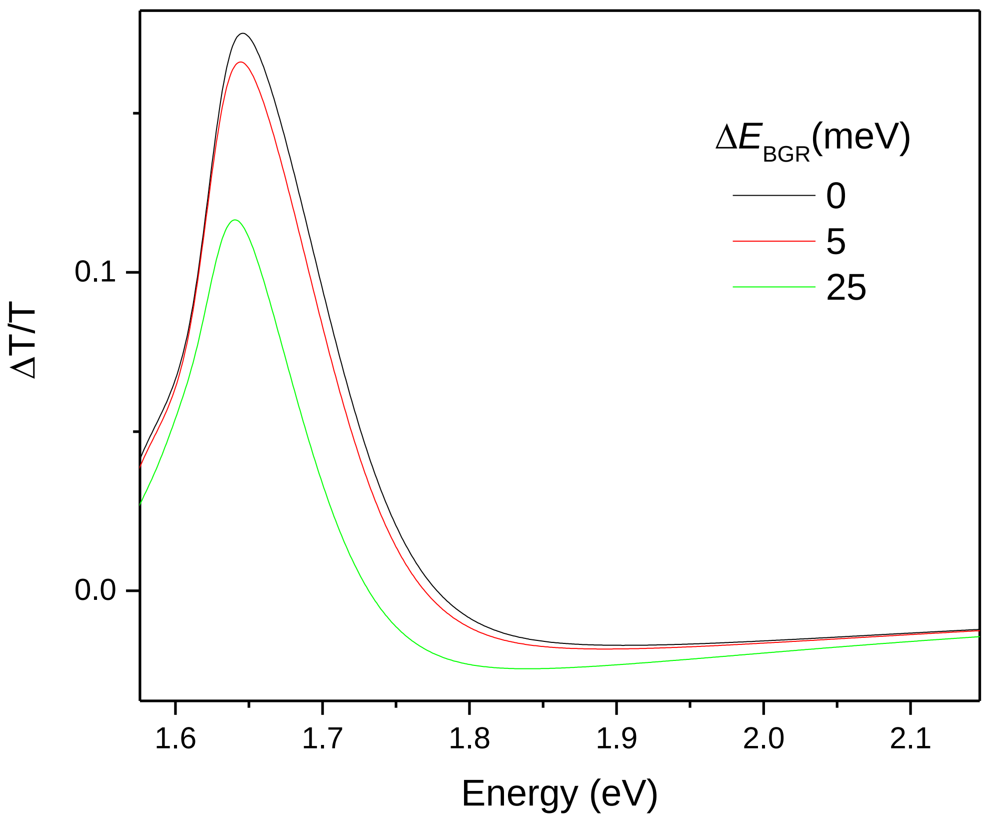

Supplementary Figure 9d

Supplementary Figure 9: Effects of varying free parameters on model TA spectra

(a) Effect of reduced effective mass on modelled transient absorption spectrum assuming no coulomb interaction. Each simulation has N = 6.4x10^{18} cm^{-3} , Γ = 0.013 eV, a = 3, C = 0.58 m^{-1}s$^{-1/2}$, ΔE_{BGR} = 5 meV. Effective masses are those stated in legend. The figure highlights how sensitive the final spectral shape (peak position and FWHM) is on m_r. **(b)** Effect of effective mass on ΔT/T kinetics. Parameters are held constant – those stated in Supplementary Figure 9a. – and effective masses are stated in legend. **(c)** Effect of effective mass asymmetry parameter, a, on modelled transient absorption spectrum. Each simulation has N = 6.4x10^{18} cm^{-3} , m_r = 0.14 m_o, Γ = 0.013 eV, C = 0.58 m^{-1}s$^{-1/2}$, ΔE_{BGR} = 5 meV. Asymmetry parameters are those stated in legend. The primary effect of a is to modify the curvature of the reflection induced

high energy negative feature. **(d)** Effect of bandgap renormalization on modelled spectral shape of transient absorption response (N = 6.4x10^{18} cm^{-3} , m_r = 0.14 m_o, Γ = 0.013 eV, C = 0.58 m^{-1}s$^{-1/2}$). Band gap shift stated in legend. At low carrier temperatures the band gap shift mostly lowers and slightly sharpens the spectral peak. The band gap shift is more apparent in the spectra at higher carrier temperatures – see Supplementary Figure 8a.

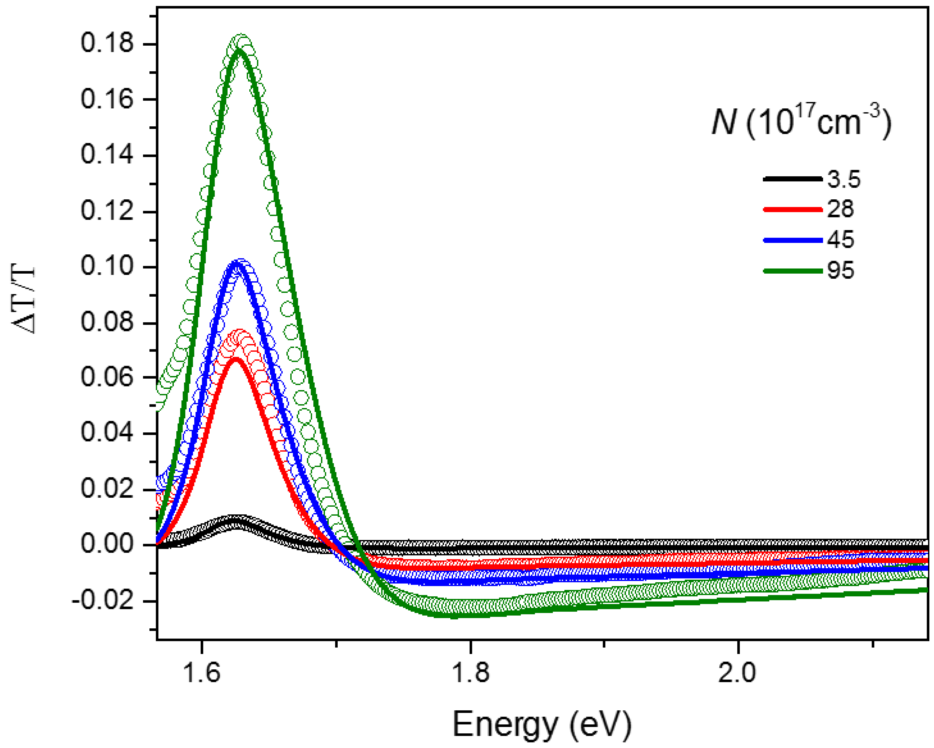

Supplementary Figure 10 Comparison of experimental TA spectra to globally fitted model for $CH_3NH_3PbI_3$ made with $PbCl_2$ precursor films.

The figure shows that the results presented in Figure 4 are generally applicable to perovskite films prepared by other techniques. In this case the experimental TA spectra (open circles) of the perovskite made with a chloride precursor agrees well with a global fit of the basic state-filling model (solid lines) for TA spectra at stated carrier densities The fitting parameters are m_r*= of 0.15 m_o, an effective mass asymmetry of a = 1.25 and an absorption constant of C = $5.6\text{x}10^{-3}$ $m^{-1}s^{-1/2}$.

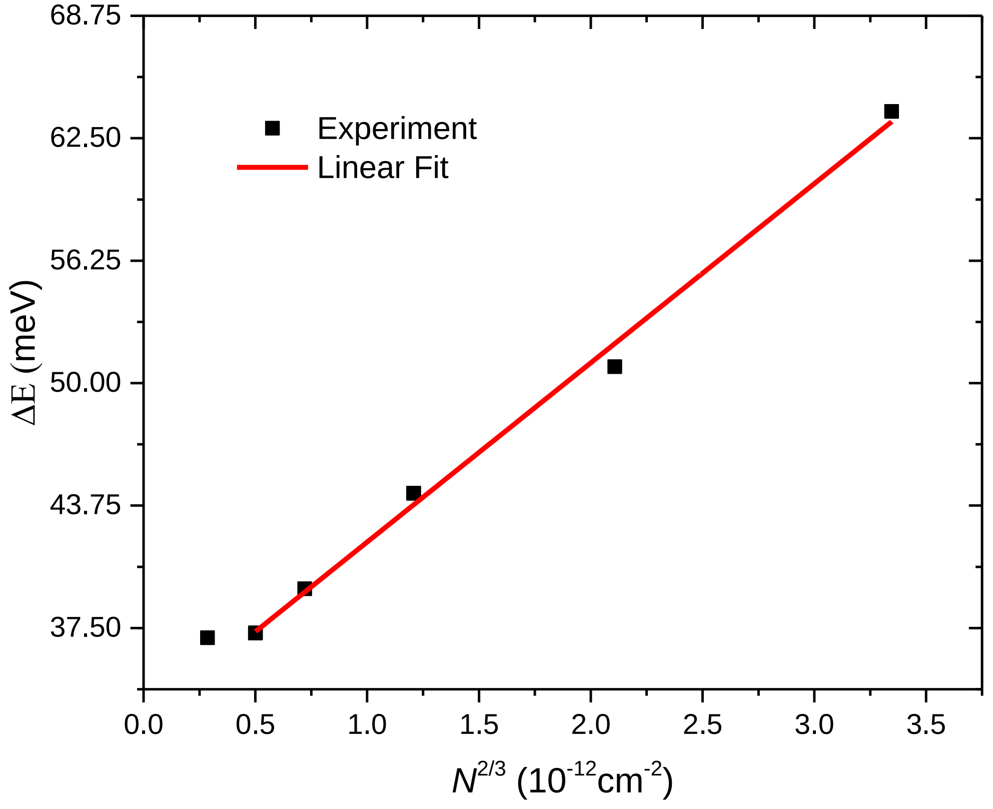

Supplementary Figure 11: **Burstein-Moss shift for reduced effective mass calculation, without refractive index changes.**

The full width at half maximum of the spectra is plotted against $N^{2/3}$. We perform an analysis as per Manser *et al.*[1] A linear fit gives a gradient consistent with a reduced effective mass of m_r = 0.38 m_o.

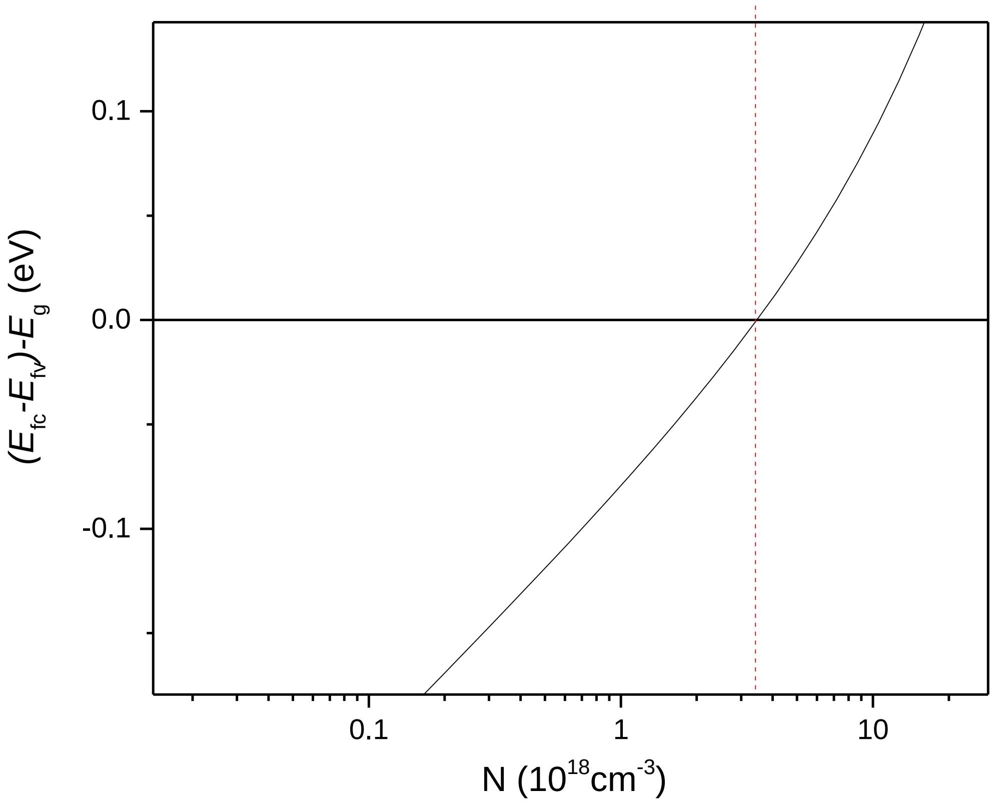

Supplementary Figure 12: Critical carrier density for optical gain

Difference in quasi-Fermi energies of conduction and valence bands minus the bandgap, versus carrier density, according to simple band-filling model with parameters described in Fig. 4 The threshold for optical gain is the point at which $(E_{fc}-E_{fv})-E_g$ becomes greater than zero. The dashed red line indicates that this transition is predicted to occur at $N \sim 2.5\mathrm{x}10^{18}$ cm^{-3}.

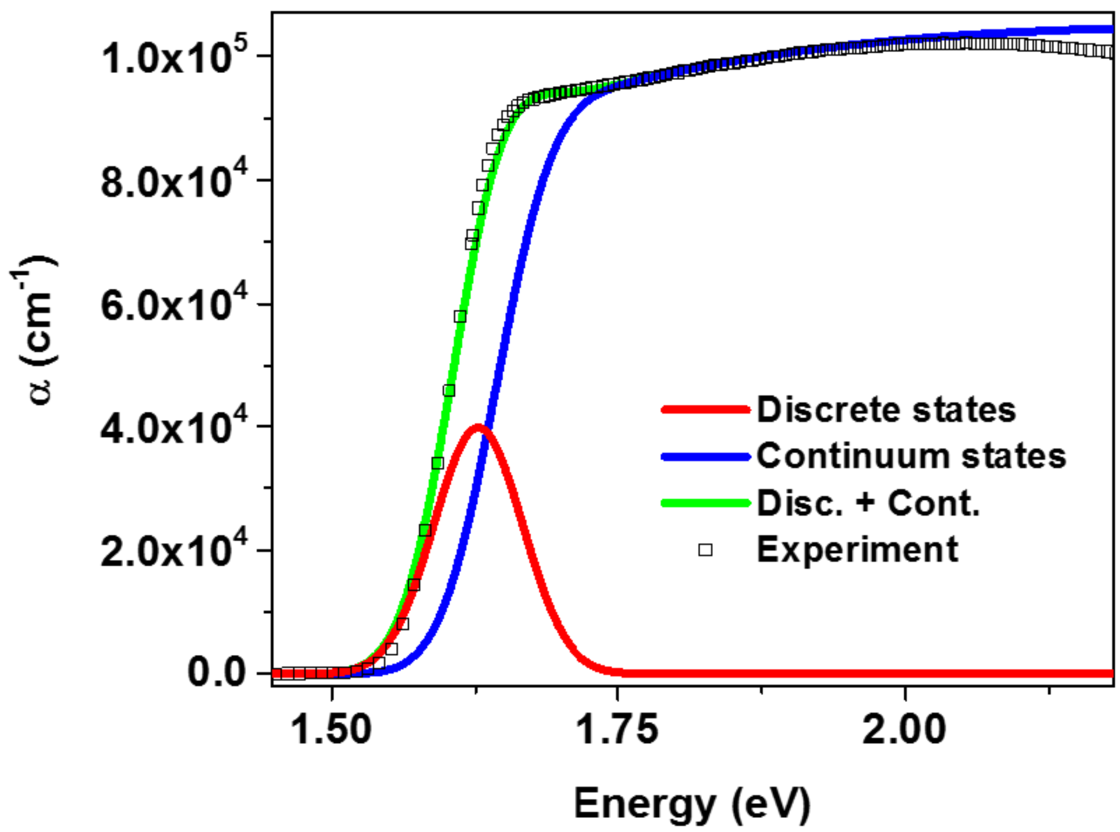

Supplementary Figure 13a

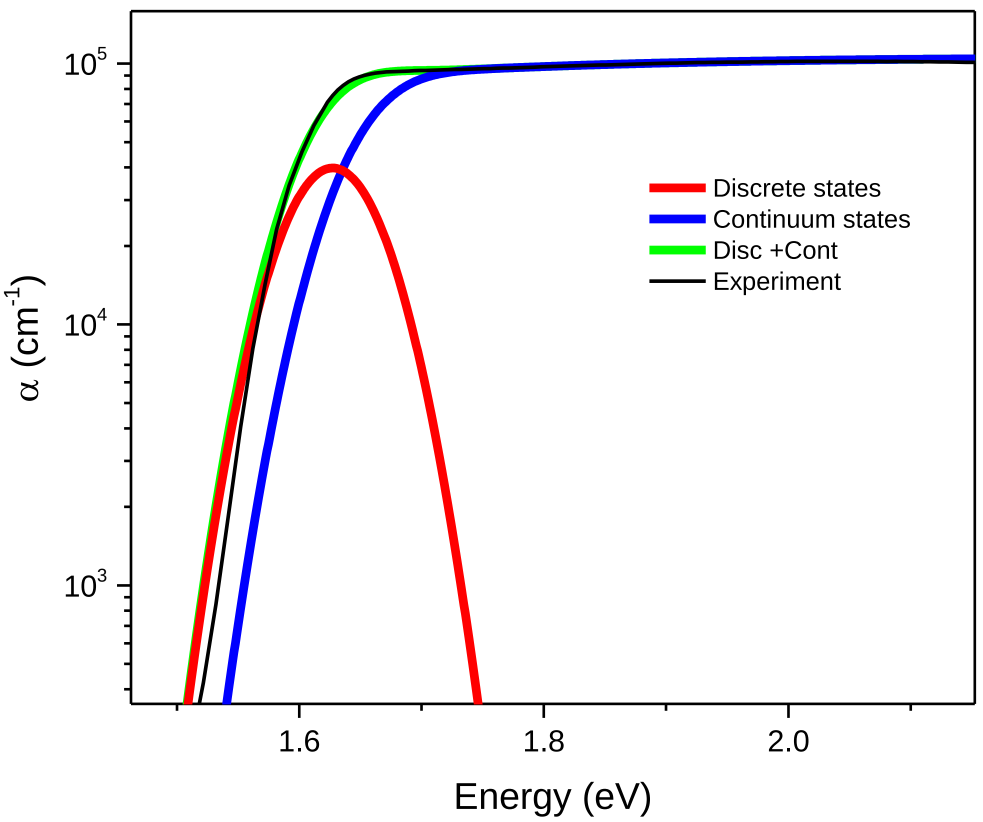

Supplementary Figure 13 b

Supplementary Figure 13: Steady state absorption of a film (65 nm) of $CH_3NH_3PbI_3$ on spectrosil. (a) The thin black squares show the absorption taken by reflection-corrected UV-vis and photothermal deflection spectroscopy (PDS). The bright green line is a fit to the experimental data according to Elliot's Wannier exciton theory, broadened with a pseudo-Voigt profile. Exciton binding energy ε_B= 17 meV, E_g = 1.645 eV, Γ = 0.045 eV. The Lorentzian portion of the broadening profile was given a weighting of 0.001. The broadening parameters were obtained by a comparison of the band tail of the model to the experimental data over three orders of magnitude (see Supplementary Figure 13b). The blue line shows the contribution to

the model spectra from continuum states, and the red line shows the contribution from discrete states (Wannier excitons). (**b)** Supplementary Figure 13a with a log scale y-axis. For nearly 3 orders of magnitude in α the band tail shows purely Gaussian (inhomogeneous) broadening. The model shows good agreement with the experimental data over this range. The PDS absorption data was spliced together with the scatter-free UV-Vis at $\alpha = 5x10^4\ cm^{-1}$.

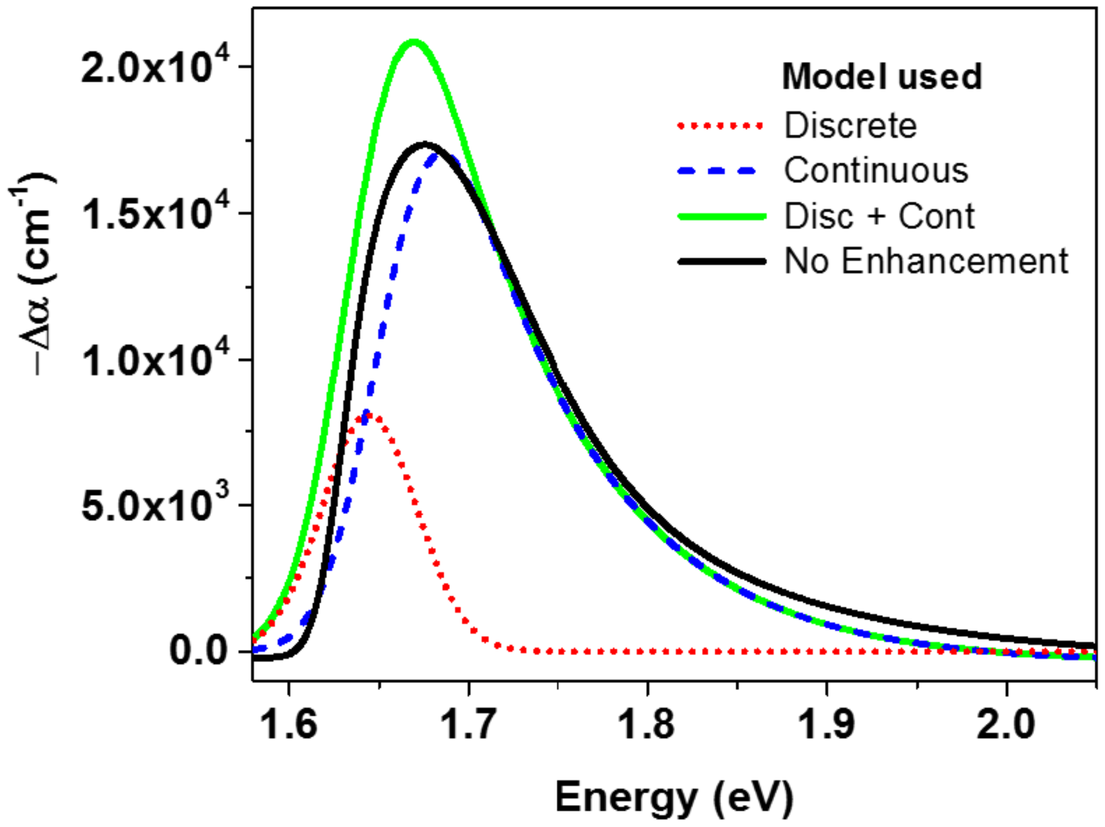

Supplementary Figure 14: Models of change in absorption coefficient due to the presence of photo-excited states.

The solid red line is a model for the change in absorption coefficient that includes band-filling effects, Coulomb enhancement and reduction in oscillator strength due to screening and/or occupation of excitonic transitions. Carrier density N = 6x10^{18} cm^{-3}, reduced mass, m_r = 0.14 m_o, ε_B= 17 meV, Γ = 0.04 eV, effective mass asymmetry a = 1.9. The red dashed line shows the contribution to this model due to change in the continuum states, and the dotted line shows the modelled contribution due to reduction of the excitonic transition. The black curve is a change in absorption coefficient with a simpler model that neglects Coulombic effects. m_r = 0.14 m_o, Γ = 0.03 eV, a = 3. We see that for higher energies, above ~1.75 eV, where the signal is dominated by band-filling and Elliot's absorption theory converges towards a simple

$\frac{1}{E}\sqrt{E - E_{\mathrm{g}}}$ dependence, the non-excitonic model broadly agrees with the model that includes a Coulomb enhancement.

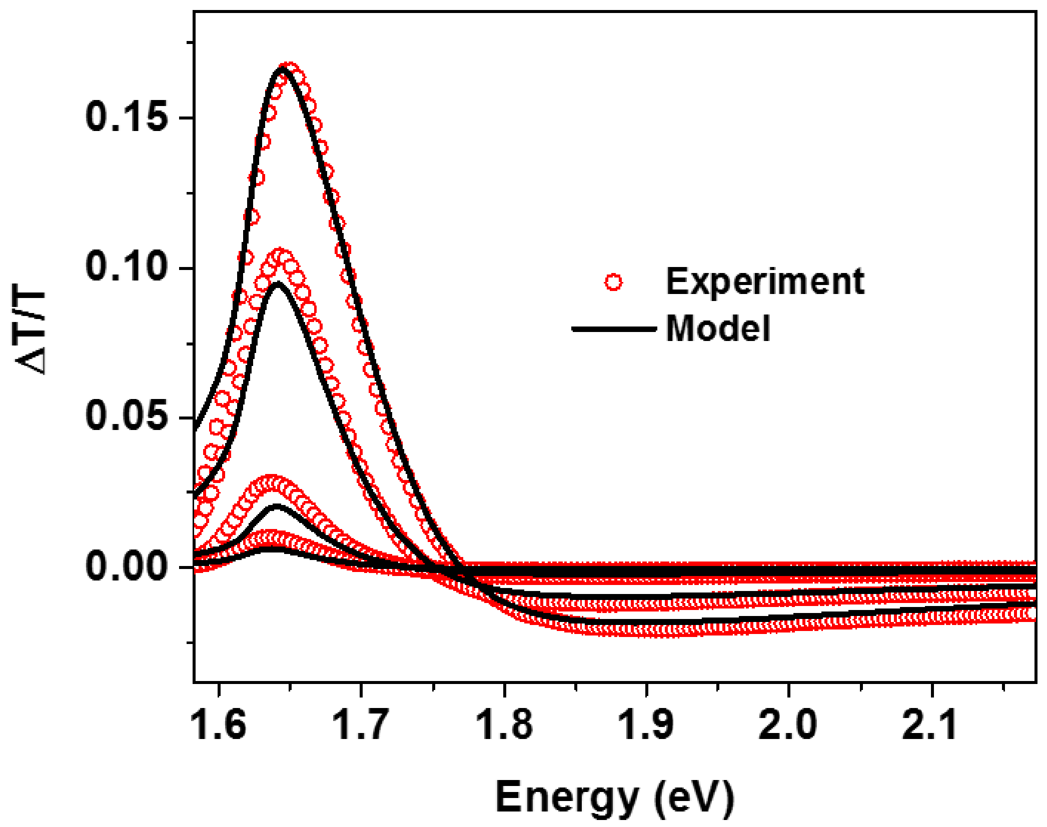

Supplementary Figure 15: Non-normalized global fit of model to experimental TA spectra. This is the same data (without normalization) as that presented in Fig. 4a. Photo-induced change in transmitted probe signal ΔT/T (open circles) for $CH_3NH_3PbI_3$ for a range of carrier densities, taken at 4 ps time delay with 2.25 eV excitation energy, incident probe angle = 90°, and probe polarization = 54.7°, Global fits of the experimental data to the model shown in Fig. 3b and outlined in the text are shown as solid lines The parameters for the model are m_r = 0.14 m_o, Γ = 0.013 eV, a = 3, C = 0.58 $m^{-1}s^{-1/2}$.

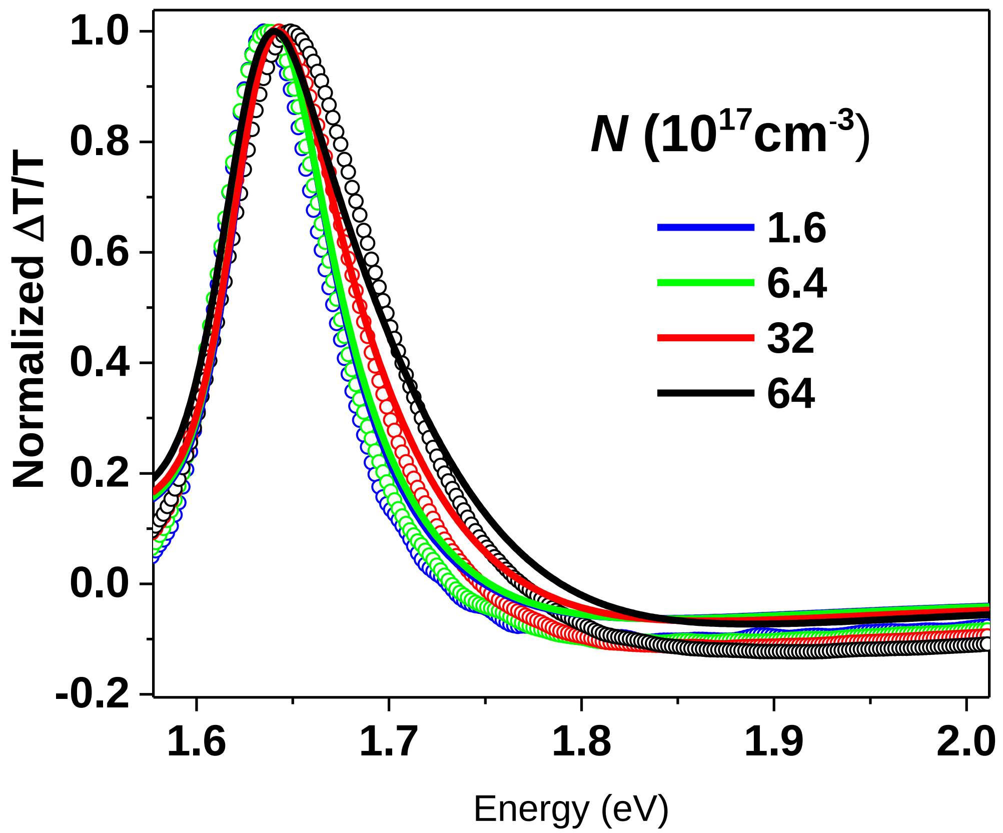

Supplementary Figure 16: Globally fitted model TA spectra including coulomb enhancement and interference effects

Normalized photo-induced change in transmitted probe signal ΔT/T (open circles) for $CH_3NH_3PbI_3$ for a range of carrier densities, taken at 4 ps time delay with 2.25 eV excitation energy, incident probe angle = 90°, and probe polarization = 54.7°, Global fits of this data for a model that includes thin film interference and Coulomb enhancement of continuum states, as described in the Supplementary Methods section, are shown as solid lines. The parameters for the model are m_r = 0.14 m_o, Γ = 0.02 eV, a = 1.3 and E_g = 1.634 eV. The model captures the Burstein-Moss spectral broadening with increasing carrier density, which is controlled by the reduced effective mass. The globally fitted reduced mass m_r = 0.14 ±0.04 m_o agrees with the

global fit of the model that does not include interference or Coulombic enhancement from Fig. 4.

Supplementary Methods

Models of carrier induced change in absorption

Supplementary Figure 13a shows the steady state absorption of $CH_3NH_3PbI_3$, taken by a combination of reflection corrected UV-Vis and photothermal deflection spectroscopy. By carefully accounting for reflection and minimising light scatter we attain a spectrum better suited for extraction of optical parameters.

We fit the spectrum using Elliot's theory of Wannier excitons in bulk inorganic semiconductors[2],[3]. We fit our broadening parameters by closely analysing the tail of the absorption for three orders of magnitude (Supplementary Figure 13b). By obtaining such a clean absorption spectrum with minimal scatter and reflection we are able to avoid an overestimation of our broadening parameters (and hence overestimation of exciton binding energy). A pseudo-Voigt profile was used initially to account for both homogeneous and inhomogeneous broadening. However, as can be seen in Supplementary Figure 10b, the Lorentzian portion of this profile was found to be small (Lorentzian weighting factor of <0.0001) and so the absorption near the band-edge must be dominated by inhomogeneous broadening and was convoluted with a simple Gaussian. We find a best fit for a small exciton binding energy, ε_B = 17 meV, with a Gaussian broadening parameter Γ = 45 meV, and band-gap of 1.645 eV.

We can extend this theory in a basic fashion to help explain carrier-induced changes in absorption coefficient, which are modelled in SupplementaryFigure 14.

Our models are based primarily on simple theories of state-filling. Our hot-carrier analysis has shown good agreement with a simple state-filling model for states near the re-normalized band-edge. Others[1,4] have also shown that, for energies away from the band-edge and for large carrier

densities, the transient absorption and photoluminescence spectra are consistent with a simple band-filling model. We thus assume a carrier dependent absorption coefficient given by [5],

$$\alpha(E,N,P) = \alpha_0(E)[f_v(E_v) - f_c(E_c)] \quad (1)$$

where $\alpha_0(E)$ is the absorption coefficient without carrier injection, N, P are the concentrations of electrons and holes, E_v, E_c, the energies of the conduction and valence bands which depend on the effective masses of electron and holes, and f_v, f_c the Fermi-Dirac distribution functions for carriers occupying either band. We estimate the quasi-Fermi levels for the electron and hole distribution functions by the Nilsson approximation[6] . We assume an effective density of states for electrons and holes given by parabolic bands, $N_{c,v} = 2(\frac{m_{e,h}k_bT}{2\pi\hbar})^{\frac{3}{2}}$, and take the perovskite on glass as being close to an intrinsic semiconductor[7], setting N=P. From the steady state absorption (Supplementary Figure 13a), we would not expect any significant band non-parabolicity until high in the band (above 1.9 eV).

Supplementary Figure 14 shows a modelled change in absorption spectra based on band-filling and an initial absorption coefficient, $\alpha_0(E)$, calculated from Elliot's theory, as described above.

The $\Delta\alpha$ curves of Supplementary Figure 14 are examples of representative spectra for a chosen set of parameters. The red, blue and green curves illustrate how the $\Delta\alpha$ spectrum can be composed of contributions from both discrete and continuum states, where their bleach is due to a reduction in oscillator strength that is modelled here as a simple occupation effect but may also be due to screening of Coulombic enhancement[8] – though we note that our attempts to model this screening by including even the smallest reduction in exciton binding energy result in simulated spectra that do not match experiment. The black curve is a model of $\Delta\alpha$ with the same reduced effective mass as the red curve, but for a model that does not include any Coulomb enhancement term or discrete excitonic states. We see that (for energies above

~1.7 eV), $\Delta\alpha$ converges for the two theories, as Elliot's absorption theory converges towards the simple $\alpha(E) = \frac{1}{E}\sqrt{E - E_{\mathrm{g}}}$ parabolic band-to-band absorption coefficient.

Photo-induced changes in refractive index

We account for the change in refractive index in our model by employing the Kramers-Kronig relations to obtain Δn from the principal integral of $\Delta\alpha$[9],

$$\Delta n(N, E) = \frac{\hbar c}{\pi} P \int_0^{\infty} \frac{\Delta\alpha(N, E')}{E'^2 - E^2} dE' \tag{2}$$

Taking the total derivative,

$$\frac{dT}{d\alpha} = \frac{\partial T}{\partial \alpha} + \frac{\partial T}{\partial n}\frac{dn}{d\alpha} \tag{3}$$

gives expressions for the transient absorption. A simple two-reflection model leads to;

$$\frac{\Delta T(E)}{T(E)} = -l\Delta\alpha(E) + 2\frac{(n_{\mathrm{g}} - n(E))}{(n(E)^2 + n(E))(n_{\mathrm{g}} + n(E))}\Delta n(E) \tag{4}$$

where l is the sample thickness and n_g is the refractive index of glass. If we include an infinite number of reflections, then the Fresnel equations combined with a Fabry-Perot analysis of thin film interference gives the following expression for the transient absorption with a normal incidence probe;

$$\frac{\Delta T(E)}{T(E)} = \frac{-((16 n_{\mathrm{g}} e^{\alpha l} l n^4 (-(n_{\mathrm{g}} - n)^2 (-1+n)^2 + e^{2\alpha l}(1+n)^2 (n_{\mathrm{g}} + n)^2))\Delta\alpha}{((n_{\mathrm{g}} - n)^2 (-1+n)^2 + e^{2\alpha l}(1+n)^2 (n_{\mathrm{g}} + n)^2 + 2e^{\alpha l}(n_{\mathrm{g}} - n)(n_{\mathrm{g}} + n)(-1+n^2)\mathrm{Cos}\left[\frac{2Eln}{\hbar c}\right])^2)} +$$

$$+\frac{16n_g e^{-\alpha l} n^4 \Delta n}{(1+n)^2(n_g+n)^2\left(1+\frac{e^{-2\alpha l}(1-n)^2(n_g-n)^2}{(1+n)^2(n_g+n)^2}-\frac{2e^{-\alpha l}(1-n)(n_g-n)\mathrm{Cos}[\frac{2Eln}{\hbar c}]}{(1+n)(n_g+n)}\right)} \quad (5)$$

A global fit of this expression for continuous states including Coulombic enhancement is shown in Supplementary Figure 16. The reduced effective mass from this analysis, $m_r = 0.14\ m_o$ agrees with the value obtained from fits of the simpler model (described in Figure 4) that does not include the possible contributions from Coulombic or interference effects.

To model the angular dependence, we must take the general case. The reflected intensity can be calculated from,

$$R = I_0(r_0^2(1+e^{-2\alpha l\mathrm{Sec}[\theta]}r_1^2r_2^2)+2e^{-2\alpha l\mathrm{Sec}[\theta]}r_1^2r_2t_1t_3+e^{-2l\alpha\mathrm{Sec}[\theta]}r_1^2t_1^2t_3^2 - 2\mathrm{Cos}[\delta]r_0(e^{-\alpha l\mathrm{Sec}[\theta]}r_0r_1r_2+e^{-\alpha l\mathrm{Sec}[\theta]}r_1t_1t_3))/(1 - 2e^{-\alpha l\mathrm{Sec}[\theta]}\mathrm{Cos}[\delta]r_1r_2+e^{-2\alpha l\mathrm{Sec}[\theta]}r_1^2r_2^2) \quad (6)$$

where $r_{0,}r_1, r_2$ are the angular and polarization dependent Fresnel equations' electric field reflection coefficients (from the first three reflections) , t_1, t_3 the transmission coefficients (from the first and third transmitted beams), θ is the angle of the probe inside the absorbing perovskite film, and δ is the phase shift difference between each interfering beam given by,

$$\delta = \frac{2nl\mathrm{E}}{\hbar c\mathrm{Cos}[\theta]} \quad (7)$$

The transmitted intensity is similarly given by,

$$T = I_0\frac{\mathrm{Cos}[\theta_2]\mathrm{Sec}[\theta_0]t_1^2t_2^2}{n_g(1-2\mathrm{Cos}[\frac{2nl\mathrm{E}}{\hbar c\mathrm{Cos}[\theta]}]r_1r_2+e^{-2\alpha l\mathrm{Sec}[\theta]}r_1^2r_2^2)} \quad (8)$$

where θ_0, θ_2 are the angles of the beams before and after they have entered the perovskite film. These angles are all calculated from Snell's law. We then obtain expressions for the curves shown in Supplementary Figure 7 by taking the partial derivatives with respect to α and n, and dividing by R+T to obtain $\frac{\Delta T}{T+R}$ and $\frac{\Delta R}{T+R}$.

An expression for the absorption coefficient of free carriers is given by,

$$\alpha_{\mathrm{fc}}(E,N) = \frac{4\pi\hbar e^2\gamma N}{\varepsilon_0 c m_{\mathrm{r}} n(E)(E^2 + \hbar^2\gamma^2)} \tag{9}$$

where e is the charge of an electron, ε_0 the vacuum permittivity, and γ is the scattering rate from carrier-carrier and carrier-phonon interactions[10]. An estimate of this scattering rate can be obtained by comparing our hot carrier cooling data to a basic expression for the carrier loss rate, assuming a dominant energy loss from LO phonons[11],

$$\langle\frac{dE}{dt}\rangle = \hbar\omega_{\mathrm{LO}}/\tau_{\mathrm{LO}}\left(e^{-\hbar\omega_{\mathrm{LO}}/k_{\mathrm{B}}T_{\mathrm{c}}} - e^{-\hbar\omega_{\mathrm{LO}}/k_{\mathrm{B}}T_{\mathrm{L}}}\right) \tag{10}$$

where τ_{LO} is the average energy loss rate, $\hbar\omega_{\mathrm{LO}}$ the LO phonon energy, T_{L} is the lattice temperature and T_{c} the effective carrier temperature. For an LO phonon energy of 13 meV – the same as lead iodide, the data fits the expression for an average energy loss constant of $\tau_{\mathrm{LO}} = 20$ fs, which we use as an estimate of γ, the scattering rate. This is in broad agreement with a theoretical calculation[11] of $\tau_{\mathrm{LO}} = 8$ fs, based on values of static and optical dielectric constants from [12]. For this value of γ, the free carrier absorption in the visible region is negligible. Even if the scattering rate were two orders of magnitude greater, the free carrier term still has little effect on the TA spectra.

Many treatments have given expressions for the expected band gap shift due to the presence and interaction of free carriers[13,14,15,16]. This band-gap renormalization is always calculated to show a weak dependence on carrier density, i.e. ΔE scales with N^q where $q < ½$. Supplementary Figure 9d shows the effect of varying the band-gap shift at a given carrier density for a carrier distribution at 300 K. For carriers at 300 K the spectral shape from our model is not significantly changed for small to moderate values (ΔE <15 meV at N = 6.4×10^{18} cm^{-3}). We find good fits to experimental data with a model of the density dependence of the band-gap renormalization shift with a phenomenological form based on III-V semiconductors[17], $\Delta E_{\mathrm{BGR}} = B(N - N_c)^{1/3}$, with N_c = 1×10^{16} cm^{-3} and B = 1.2×10^4 eV $m^{3/2}$.

Supplementary References

1. Manser, J. S. & Kamat, P. V. Band filling with free charge carriers in organometal halide perovskites. *Nat. Photonics* (2014). doi:10.1038/nphoton.2014.171

2. Elliot, R. J. Intensity of Optical Absorption by Excitons. *Phys. Rev.* **108,** (1957).

3. Sturge, M. D. Optical Absorption of Gallium Arsenide between 0.6 and 2.75 eV. *Phys. Rev.* **127,** 768–773 (1962).

4. Deschler, F. *et al.* High Photoluminescence Efficiency and Optically-Pumped Lasing in Solution-Processed Mixed Halide Perovskite Semiconductors. *J. Phys. Chem. Lett.* 140324121627005 (2014). doi:10.1021/jz5005285

5. Trevor S. Moss, Geoffrey John Burrell, Brian Ellis. *Semiconductor Opto-Electronics*. (Butterworths, 1973).

6. Nilsson, N. G. Empirical approximations for the Fermi energy in a semiconductor with parabolic bands. *Appl. Phys. Lett.* **33,** 653 (1978).

7. Leijtens, T. *et al.* Electronic Properties of Meso-Superstructured and Planar Organometal Halide Perovskite Films : Charge Trapping, Photodoping, amd Carrier Mobility. *ACS Nano* (2014).

8. Haug, H. & Schmitt-Rink, S. Basic mechanisms of the optical nonlinearities of semiconductors near the band edge. *J. Opt. Soc. Am. B* **2,** 1135 (1985).

9. Ree, E. Y. F., Ctron, E. L. E. & Stern, F. Dispersion of the Index of Refraction Near the Absorption Edge of Semiconductors. *Phys. Rev.* **776,** (1957).

10. Yu, P. Y. & Cardona, M. *Fundamentals of Semiconductors: Physics and Materials Properties*. (Springer, 2001).

11. Shah, J. *Ultrafast Spectroscopy of Semiconductors and Semiconductor Nanostructures*. (Springer, 1999).

12. Lin, Q. *et al.* Electro-optics of perovskite solar cells. *Nat. Photonics* **9,** 106–112 (2014).

13. Banyai, L. & Koch, S. W. A simple theory for the effects of plasma screening on the optical spectra of highly excited semiconductors. *Zeitschrift fur Phys. B Condens. Matter* **63,** 283–291 (1986).

14. Versteegh, M. a. M., Kuis, T., Stoof, H. T. C. & Dijkhuis, J. I. Ultrafast screening and carrier dynamics in ZnO: Theory and experiment. *Phys. Rev. B* **84,** 035207 (2011).

15. Schmid, P. E. Optical Absorption in Heavily Doped Silicon. *Phys. Rev. B* **23,** (1981).

16. Lu, J. G. *et al.* Carrier concentration dependence of band gap shift in n-type ZnO:Al films. *J. Appl. Phys.* **101,** 083705 (2007).

17. Bennett, B. R., Soref, R. A., Member, S. & Alamo, J. A. D. E. L. Carrier-Induced Change in Refractive Index of InP , GaAs , and InGaAsP. *IEEE J. Quantum Electron.* **26,** (1990).
